# Conformist social learning leads to self-organised prevention against adverse bias in risky decision making

**DOI:** 10.7554/eLife.75308

**Published:** 2022-05-10

**Authors:** Wataru Toyokawa, Wolfgang Gaissmaier

**Affiliations:** 1 Department of Psychology, University of Konstanz Konstanz Germany; 2 Centre for the Advanced Study of Collective Behaviour, University of Konstanz, Konstanz Germany; University of California, Irvine United States; Brown University United States

**Keywords:** social learning, conformity, reinforcement learning, hot stove effect, risky decision making, collective behaviour, Human

## Abstract

Given the ubiquity of potentially adverse behavioural bias owing to myopic trial-and-error learning, it seems paradoxical that improvements in decision-making performance through conformist social learning, a process widely considered to be bias amplification, still prevail in animal collective behaviour. Here we show, through model analyses and large-scale interactive behavioural experiments with 585 human subjects, that conformist influence can indeed promote favourable risk taking in repeated experience-based decision making, even though many individuals are systematically biased towards adverse risk aversion. Although strong positive feedback conferred by copying the majority’s behaviour could result in unfavourable informational cascades, our differential equation model of collective behavioural dynamics identified a key role for increasing exploration by negative feedback arising when a weak minority influence undermines the inherent behavioural bias. This ‘collective behavioural rescue’, emerging through coordination of positive and negative feedback, highlights a benefit of collective learning in a broader range of environmental conditions than previously assumed and resolves the ostensible paradox of adaptive collective behavioural flexibility under conformist influences.

## eLife digest

When it comes to making decisions, like choosing a restaurant or political candidate, most of us rely on limited information that is not accurate enough to find the best option. Considering others’ decisions and opinions can help us make smarter choices, a phenomenon called “collective intelligence”.

Collective intelligence relies on individuals making unbiased decisions. If individuals are biased toward making poor choices over better ones, copying the group’s behavior may exaggerate biases. Humans are persistently biased. To avoid repeated failure, humans tend to avoid risky behavior. Instead, they often choose safer alternatives even when there might be a greater long-term benefit to risk-taking. This may hamper collective intelligence.

Toyokawa and Gaissmaier show that learning from others helps humans make better decisions even when most people are biased toward risk aversion. The experiments first used computer modeling to assess the effect of individual bias on collective intelligence. Then, Toyokawa and Gaissmaier conducted an online investigation in which 185 people performed a task that involved choosing a safer or risker alternative, and 400 people completed the same task in groups of 2 to 8. The online experiment showed that participating in a group changed the learning dynamics to make information sampling less biased over time. This mitigated people’s tendency to be risk-averse when risk-taking is beneficial.

The model and experiments help explain why humans have evolved to learn through social interactions. Social learning and the tendency of humans to conform to the group’s behavior mitigates individual risk aversion. Studies of the effect of bias on individual decision-making in other circumstances are needed. For example, would the same finding hold in the context of social media, which allows individuals to share unprecedented amounts of sometimes incorrect information?

## Introduction

Collective intelligence, a self-organised improvement of decision making among socially interacting individuals, has been considered one of the key evolutionary advantages of group living (Harrison et al., 2001; Krause and Ruxton, 2002; Sumpter, 2005; Ward and Zahavi, 1973). Although what information each individual can access may be a subject of uncertainty, information transfer through the adaptive use of social cues filters such ‘noises’ out (Laland, 2004; Rendell et al., 2010), making individual behaviour on average more accurate (Hastie and Kameda, 2005; King and Cowlishaw, 2007; Simons, 2004). Evolutionary models (Boyd and Richerson, 1985; Kandler and Laland, 2013; Kendal et al., 2005) and empirical evidence (Toyokawa et al., 2014; Toyokawa et al., 2019) have both shown that the benefit brought by the balanced use of both socially and individually acquired information is usually larger than the cost of possibly creating an alignment of suboptimal behaviour among individuals by herding (Bikhchandani et al., 1992; Giraldeau et al., 2002; Raafat et al., 2009). This prediction holds as long as individual trial-and-error learning leads to higher accuracy than merely random decision making (Efferson et al., 2008). Copying a common behaviour exhibited by many others is adaptive if the output of these individuals is expected to be better than uninformed decisions.

However, both humans and non-human animals suffer not only from environmental noise but also commonly from systematic biases in their decision making (e.g. Harding et al., 2004; Hertwig and Erev, 2009; Real, 1981; Real et al., 1982). Under such circumstances, simply aggregating individual inputs does not guarantee collective intelligence because a majority of the group may be biased towards suboptimization. A prominent example of such a potentially suboptimal bias is risk aversion that emerges through trial-and-error learning with adaptive information-sampling behaviour (Denrell, 2007; March, 1996). Because it is a robust consequence of decision making based on learning (Hertwig and Erev, 2009; Yechiam et al., 2006; Weber, 2006; March, 1996), risk aversion can be a major constraint of animal behaviour, especially when taking a high-risk high-return behavioural option is favourable in the long run. Therefore, the ostensible prerequisite of collective intelligence, that is, that individuals should be unbiased and more accurate than mere chance, may not always hold. A theory that incorporates dynamics of trial-and-error learning and the learnt risk aversion into social learning is needed to understand the conditions under which collective intelligence operates in risky decision making.

Given that behavioural biases are omnipresent and learning animals rarely escape from them, it may seem that social learning, especially the ‘copy-the-majority’ behaviour (aka, ‘conformist social learning’ or ‘positive frequency-based copying’; Laland, 2004), whereby the most common behaviour in a group is disproportionately more likely to be copied (Boyd and Richerson, 1985), may often lead to maladaptive herding, because recursive social interactions amplify the common bias (i.e. a positive feedback loop; Denrell and Le Mens, 2007; Denrell and Le Mens, 2017; Dussutour et al., 2005; Raafat et al., 2009). Previous studies in humans have indeed suggested that individual decision-making biases are transmitted through social influences (Chung et al., 2015; Bault et al., 2011; Suzuki et al., 2016; Shupp and Williams, 2008; Jouini et al., 2011; Moussaïd et al., 2015). Nevertheless, the collective improvement of decision accuracy through simple copying processes has been widely observed across different taxa (Sasaki and Biro, 2017; Seeley et al., 1991; Alem et al., 2016; Sumpter, 2005; Harrison et al., 2001), including the very species known to exhibit learnt risk-taking biases, such as bumblebees (Real, 1981; Real et al., 1982), honeybees (Drezner-Levy and Shafir, 2007), and pigeons (Ludvig et al., 2014). Such observations may indicate, counter-intuitively, that social learning may not necessarily trap animal groups in suboptimization even when most of the individuals are suboptimally biased.

In this paper, we propose a parsimonious computational mechanism that accounts for the emerging improvement of decision accuracy among suboptimally risk-aversive individuals. In our agent-based model, we allow our hypothetical agents to compromise between individual trial-and-error learning and the frequency-based copying process, that is, a balanced reliance on social learning that has been repeatedly supported in previous empirical studies (e.g. Deffner et al., 2020; McElreath et al., 2005; McElreath et al., 2008; Toyokawa et al., 2017; Toyokawa et al., 2019). This is a natural extension of some previous models that assumed that individual decision making was regulated fully by others’ beliefs (Denrell and Le Mens, 2007; Denrell and Le Mens, 2017). Under such extremely strong social influence, exaggeration of individual bias was always the case because information sampling was always directed towards the most popular alternative, often resulting in a mismatch between the true environmental state and what individuals believed (’collective illusion’; Denrell and Le Mens, 2017). By allowing a mixture of social and asocial learning processes within a single individual, the emergent collective behaviour is able to remain flexible (Aplin et al., 2017; Toyokawa et al., 2019), which may allow groups to escape from the suboptimal behavioural state.

We focused on a repeated decision-making situation where individuals updated their beliefs about the value of behavioural alternatives through their own action–reward experiences (experience-based task). Experience-based decision making is widespread in animals that learn in a range of contexts (Hertwig and Erev, 2009). The time-depth interaction between belief updating and decision making may create a non-linear relationship between social learning and individual behavioural biases (Biro et al., 2016), which we hypothesised is key in improving decision accuracy in self-organised collective systems (Harrison et al., 2001; Sumpter, 2005).

In the study reported here, we firstly examined whether a simple form of conformist social influence can improve collective decision performance in a simple multi-armed bandit task using an agent-based model simulation. We found that promotion of favourable risk taking can indeed emerge across different assumptions and parameter spaces, including individual heterogeneity within a group. This phenomenon occurs thanks, apparently, to the non-linear effect of social interactions, namely, *collective behavioural rescue*. To disentangle the core dynamics behind this ostensibly self-organised process, we then analysed a differential equation model representing approximate population dynamics. Combining these two theoretical approaches, we identified that it is a combination of positive and negative feedback loops that underlies collective behavioural rescue, and that the key mechanism is a promotion of information sampling by modest conformist social influence.

Finally, to investigate whether the assumptions and predictions of the model hold in reality, we conducted a series of online behavioural experiments with human participants. The experimental task was basically a replication of the task used in the agent-based model described above, although the parameters of the bandit tasks were modified to explore wider task spaces beyond the simplest two-armed task. Experimental results show that the human collective behavioural pattern was consistent with the theoretical prediction, and model selection and parameter estimation suggest that our model assumptions fit well with our experimental data. In sum, we provide a general account of the robustness of collective intelligence even under systematic risk aversion and highlight a previously overlooked benefit of conformist social influence.

## Results

### The decision-making task

**Figure 1.**
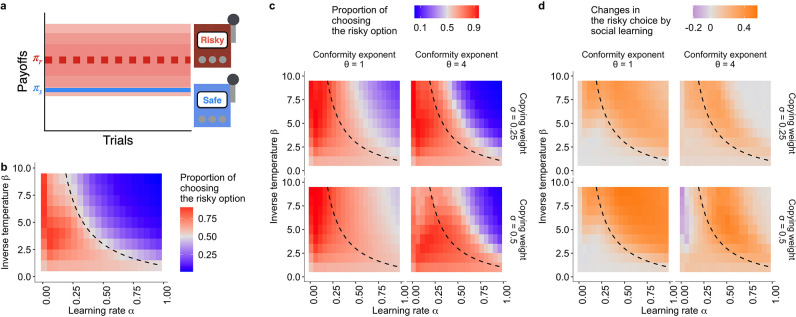
Mitigation of suboptimal risk aversion by social influence. (**a**) A schematic diagram of the task. A safe option provides a constant reward $\pi_{s}=1$ whereas a risky option provides a reward randomly drawn from a Gaussian distribution with mean μ=1.5 and s.d.=1. (**b, c**): The emergence of suboptimal risk aversion (the hot stove effect) depending on a combination of the reinforcement learning parameters; (**b**): under no social influence (i.e. the copying weight σ=0), and (**c**): under social influences with different values of the conformity exponents θ and copying weights σ. The dashed curve is the asymptotic equilibrium at which asocial learners are expected to end up choosing the two alternatives with equal likelihood (i.e. $P_{r,t\to \infty }=0.5$), which is given analytically by β=(2-α)/α(Denrell, 2007). The coloured background is a result of the agent-based simulation with total trials T=150 and group size N=10, showing the average proportion of choosing the risky option in the second half of the learning trials $P_{r,t>75}>0.5$ under a given combination of the parameters. (**d**): The differences between the mean proportion of risk aversion of asocial learners and that of social learners, highlighting regions in which performance is improved (orange) or undermined (purple) by social learning.

The minimal task that allowed us to study both learnt risk aversion and conformist social learning was a two-armed bandit task where one alternative provided certain payoffs $\pi_{s}$ constantly (safe option s) and the other alternative provided a range of payoffs stochastically, following a Gaussian distribution $\pi_{r}∼N(\mu ,s.d.)$ (risky option r; Figure 1a). Unless otherwise stated, we followed the same task setup as Denrell, 2007, who mathematically derived the condition under which individual reinforcement learners would exhibit risk aversion. In the main analysis, we focus on the case where the risky alternative had a higher mean payoff than the safe alternative (i.e. producing more payoffs on average in the long run; positive risk premium [positive RP]), meaning that choosing the risky alternative was the optimal strategy for a decision maker to maximise accumulated payoffs. Unless otherwise stated, the total number of decision-making trials (time horizon) was set to T=150 in the main simulations described below.

**Figure 1—figure supplement 1.**
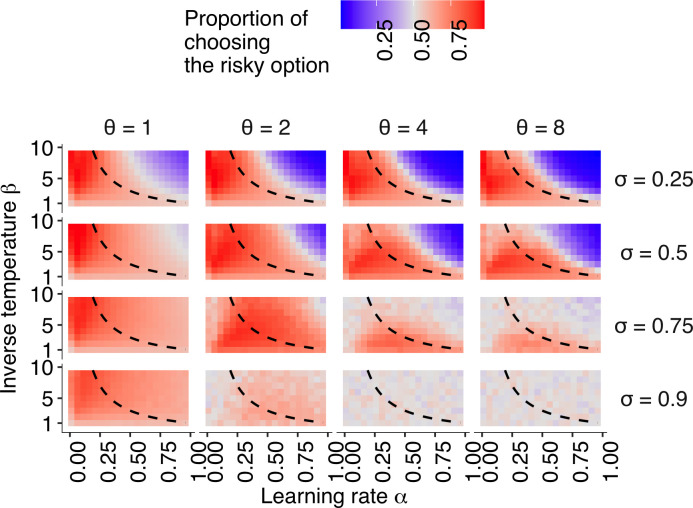
The simulation result with a wider parameter space. The effect of the relationship between individual learning rate (α) and individual inverse temperature (β) across the different combinations of social learning parameters on the mean proportion of choosing the risky alternative in the second half of the trials of the two-armed bandit task described in Figure 1 in the main text. The dashed curves give a set of parameter combinations with which asocial learners are expected to choose the risky alternative in the same proportion as they choose the safe alternative (i.e. $P_{r}^{⋆}=0.5$) in the infinite time horizon T→∞, given by β=(2-α)/α.

**Figure 1—figure supplement 2.**
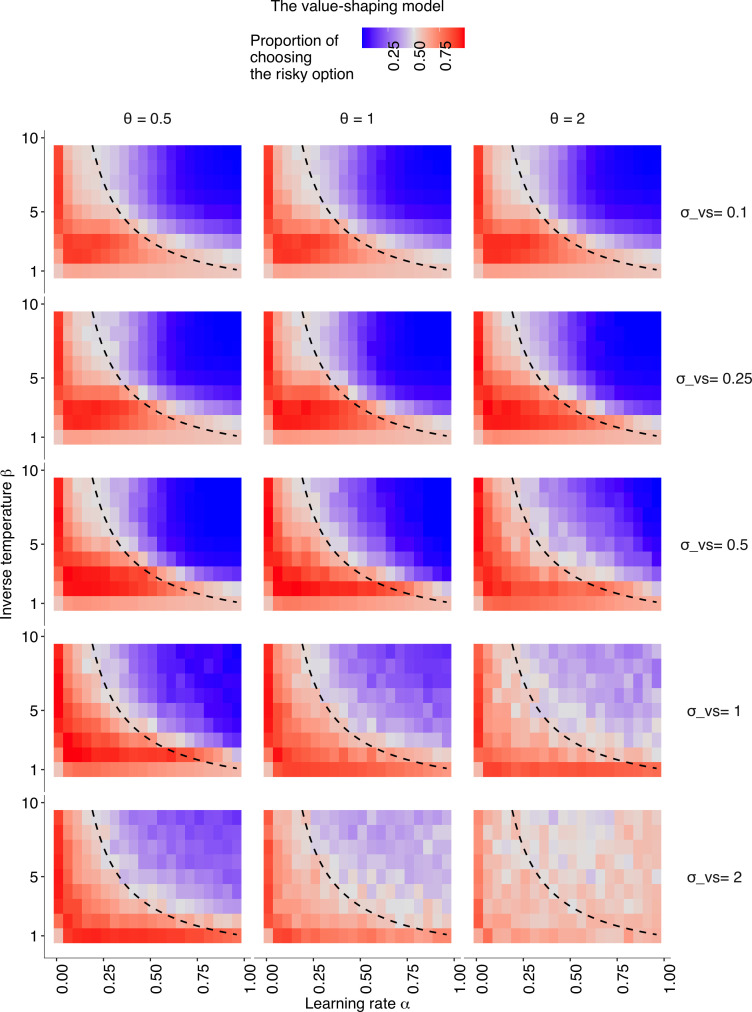
The results of the value-shaping social influence model. The relationships between individual learning rate (α) and individual inverse temperature (β) across different combinations of social learning parameters. The coloured background shows the average proportion of choosing the risky option in the second half of the learning trials $P_{r,t>75}>0.5$. Different social learning weights ($\sigma_{v\,s}$) are shown from top to bottom ($\sigma_{v\,s}\in \left\{0,0.1,0.25,0.5,1,2\right\}$). Different conformity exponents are shown from left to right (θ∈{0.5,1,2}). The dashed curve is the asymptotic equilibrium at which asocial learners are expected to end up choosing both alternatives with equal likelihood (i.e. $P_{r}^{⋆}=0.5$), given by β=(2-α)/α.

**Figure 1—figure supplement 3.**
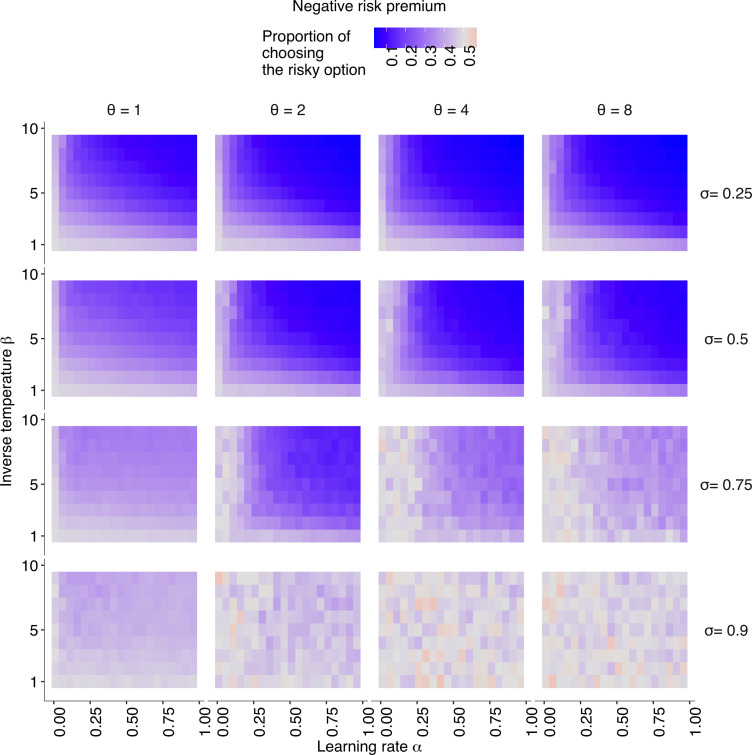
The simulation result with the negative risk premium. The relationships between individual learning rate (α) and individual inverse temperature (β) across different combinations of social learning parameters. The coloured background shows the average proportion of choosing the risky option in the second half of the learning trials $P_{r,t>75}>0.5$. Different social learning weights (σ) are shown from top to bottom (σ∈{0,0.25,0.5,0.75,0.9}). Different conformity exponents are shown from left to right (θ∈{1,2,4,8}). The risk premium is negativeμ=-0.5.

**Figure 1—figure supplement 4.**
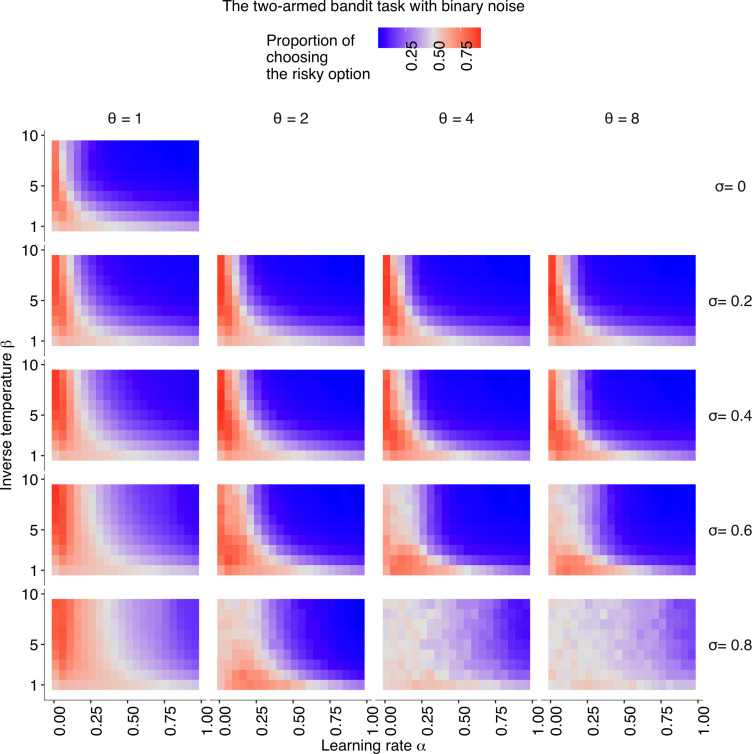
The simulation result with the Bernoulli noise distribution. The relationships between individual learning rate (α) and individual inverse temperature (β) across different combinations of social learning parameters. The coloured background shows the average proportion of choosing the risky option in the second half of the learning trials $P_{r,t>75}>0.5$. Different social learning weights (σ) are shown from top to bottom (σ∈{0,0.2,0.4,0.6,0.8}). Different conformity exponents are shown from left to right (θ∈{1,2,4,8}). The binary payoff distribution was used where the safe alternative always provides $\pi_{s}=1$ while the risky alternative provides either a 70% chance of $\pi_{r}=0$ or a 30% chance of $\pi_{r}=5$ . The risk premium was 1.5.

**Figure 1—figure supplement 5.**
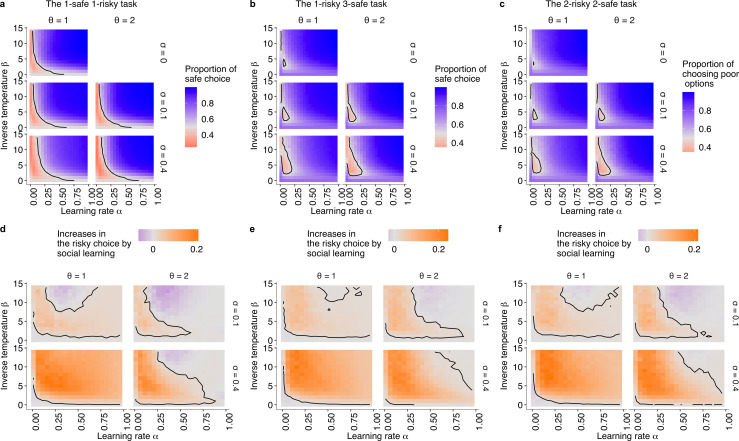
The simulation results under the positive risk premium experimental setups (a,d: the 1-risky-1-safe; b,e: the 1-risky-3-safe; c,f: the 2-risky-2-safe). The relationships between individual learning rate (α) and individual inverse temperature (β) across different combinations of social learning parameters. (a–c): The coloured background shows the average proportion of choosing the risky option in the second half of the learning trials ($P_{r,t>75}>0.5$) under social influences with different values of the conformity exponents θ and copying weights σ. The dashed curve is the asymptotic equilibrium at which asocial learners are expected to end up choosing the two alternatives with equal likelihood (i.e. $P_{r}=0.5$). (d–f): The differences between the mean proportion of risk aversion of asocial learners and that of social learners, highlighting regions in which performance is improved (that is, risk-seeking increases; orange) or undermined (that is, risk-aversion is amplified; purple) by social learning.

**Figure 1—figure supplement 6.**
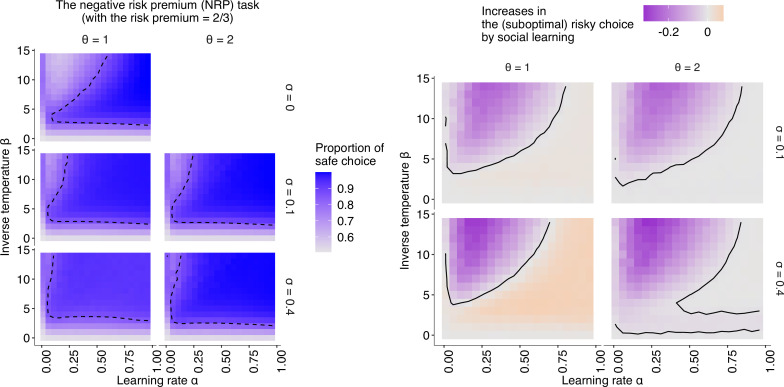
The simulation results under the negative risk premium experimental setup. The relationships between individual learning rate (α) and individual inverse temperature (β) across different combinations of social learning parameters. (left): The coloured background shows the average proportion of choosing the (optimal) safe option in the second half of the learning trials under social influences with different values of the conformity exponents θ and copying weights σ. The dashed curve shows the proportion of choosing the safe option at $P_{s}=0.85$. (right): The differences between the mean proportion of risk aversion of asocial learners and that of social learners, highlighting regions in which (suboptimal) risk-seeking increases (orange) and (optimal) risk-aversion increases (purple) by social learning.

To maximise one’s own long-term individual profit under such circumstances, it is crucial to strike the right balance between exploiting the option that has seemed better so far and exploring the other options to seek informational gain. Because of the nature of adaptive information sampling under such exploration–exploitation trade-offs, lone decision makers often end up being risk averse, trying to reduce the chance of further failures once the individual has experienced an unfavourable outcome from the risky alternative (March, 1996; Denrell, 2007; Hertwig and Erev, 2009), a phenomenon known as the *hot stove effect*. Within the framework of this task, risk aversion is suboptimal in the long run if the risk premium is positive (Denrell and March, 2001).

### The baseline model

For the baseline asocial reinforcement learning, we assumed a standard, well-established model that is a combination of the Rescorla–Wagner learning rule and softmax decision making (Sutton and Barto, 2018, see Materials and methods for the full details). There are two parameters, a *learning rate* (α) and an *inverse temperature* (β). The larger the α, the more weight is given to recent experiences, making the agent’s belief update more myopic. The parameter β regulates how sensitive the choice probability is to the belief about the option’s value (i.e. controlling the proneness to explore). As β→0, the softmax choice probability approximates to a random choice (i.e. highly explorative). Conversely, if β→+∞, it asymptotes to a deterministic choice in favour of the option with the highest subjective value (i.e. highly exploitative).

**Figure 2.**
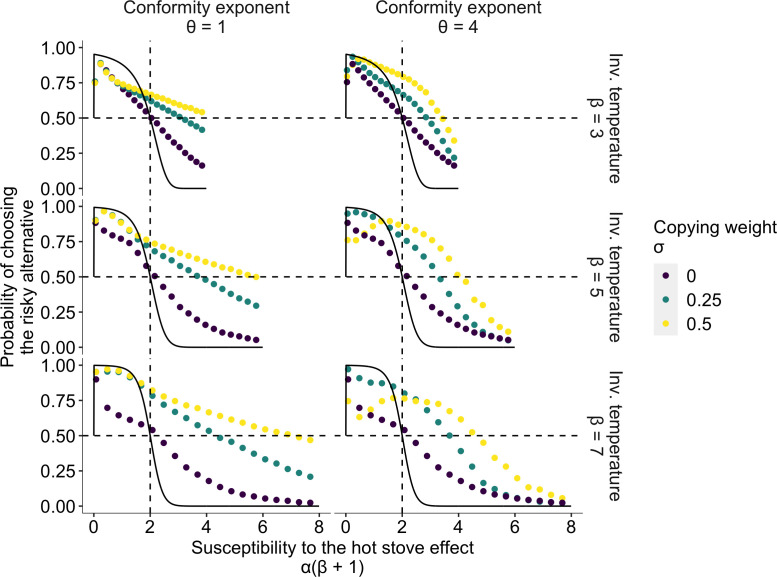
The effect of social learning on average decision performance. The *x* axis is a product of two reinforcement learning parameters α⁢(β+1), namely, the susceptibility to the hot stove effect. The *y* axis is the mean probability of choosing the optimal risky alternative in the last 75 trials in a two-armed bandit task whose setup was the same as in Figure 1. The black solid curve is the analytical prediction of the asymptotic performance of individual reinforcement learning with infinite time horizon T→+∞ (Denrell, 2007). The analytical curve shows a choice shift emerging at α⁢(β+1)=2; that is, individual learners ultimately prefer the safe to the risky option in the current setup of the task when α(β+1)>2. The dotted curves are mean results of agent-based simulations of social learners with two different mean values of the copying weight σ∈{0.25,0.5} (green and yellow, respectively) and asocial learners with σ=0 (purple). The difference between the agent-based simulation with σ=0 and the analytical result was due to the finite number of decision trials in the simulation, and hence, the longer the horizon, the closer they become (Figure 2—figure supplement 1). Each panel shows a different combination of the inverse temperature β and the conformity exponent θ.

Varying these two parameters systematically, it is possible to see under what conditions trial-and-error learning leads individuals to be risk averse (Figure 1b). Suboptimal risk aversion becomes prominent when value updating in learning is myopic (i.e. when α is large) or action selection is exploitative (i.e. when β is large) or both (the blue area of Figure 1b). Under such circumstances, the hot stove effect occurs (Denrell, 2007): Experiences of low-value payoffs from the risky option tend to discourage decision makers from further choosing the risky option, trapping them in the safe alternative. In sum, whenever the interaction between the two learning parameters α⁢(β+1) exceeds a threshold value, which was 2 in the current example, decision makers are expected to become averse to the risky option (the black solid lines in Figure 2). The hot stove effect is known to emerge in a range of model implementations and has been widely observed in previous human experiments (March, 1996; Denrell, 2007; Hertwig and Erev, 2009).

**Figure 2—figure supplement 1.**
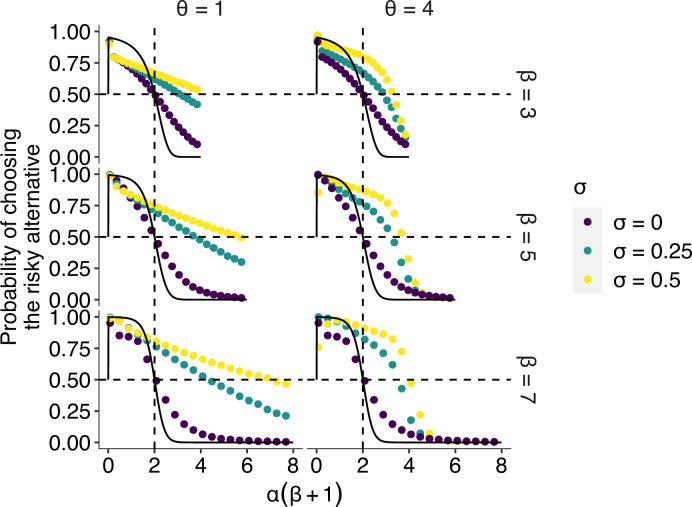
The effect of social learning on the average decision performance on the longer time horizon. The *x* axis is an interaction of two reinforcement learning parameters α⁢(β+1), that is, the susceptibility to the hot stove effect. The *y* axis is the mean probability of choosing the optimal risky alternative in the last 75 trials in the two-armed bandit task whose setup was the same as in Figures 1 and 2 in the main text (i.e. μ=0.5, s.d. = 1) except for the longer time horizon T=1075 compared to the time horizon used in the main text (T=150). The dotted curves are the mean result of agent-based simulations of groups of social learners with two different mean values of the copying weight σ∈{0.25,0.5} or individual learners with σ=0. Each panel shows a different combination of the inverse temperature β and the conformity exponent θ. The black solid curve is the theoretical benchmark where individual reinforcement learners were expected to asymptote with T→+∞. Compared to Figure 2 in the main text, individual learners got closer to the benchmark. On the other hand, the performance of social learners remained deviated from the benchmark, suggesting that social influence had a qualitative impact on the course of learning and decision making, rather than merely slowing down approaching the equilibrium of individual learning.

**Figure 2—figure supplement 2.**
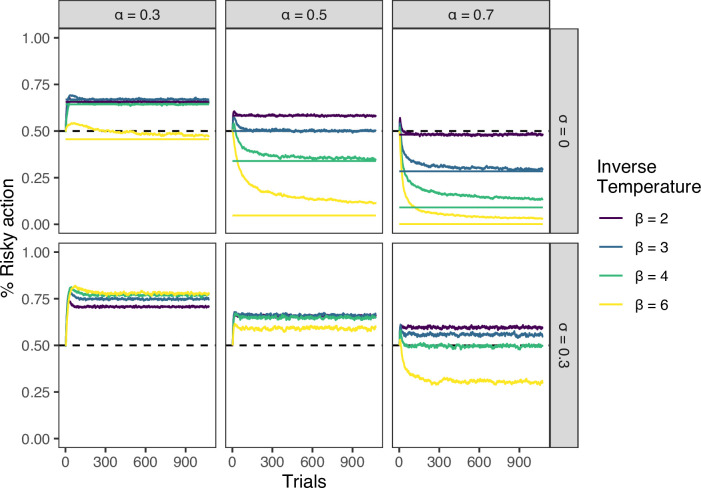
The effect of social learning on the time evolution of decision performance. The *x* axis is the number of trials. The *y* axis is the mean proportion of choosing the optimal risky alternative. Each colour shows a different β. For the asocial learning condition (i.e. σ=0), the analytical benchmark to which reinforcement learners asymptote is shown as a horizontal line. Conformity exponent θ was 2. Group size was 8. The simulation was repeated 1000 times for each combination of parameters. Compared to asocial learning cases, social learning (σ=0.3) qualitatively alters the course of learning, rather than just speeding up or slowing down learning.

### The conformist social influence model

We next considered a collective learning situation in which a group of multiple individuals perform the task simultaneously and individuals can observe others’ actions. We assumed a simple frequency-based social cue specifying distributions of individual choices (McElreath et al., 2005; McElreath et al., 2008; Toyokawa et al., 2017; Toyokawa et al., 2019; Deffner et al., 2020). We assumed that individuals could not observe others’ earnings, ensuring that they could not sample information about payoffs being no longer available because of their own choice (i.e. forgone payoffs; Denrell, 2007; Yechiam and Busemeyer, 2006).

A realised payoff was independent of others’ decisions and was drawn solely from the payoff probability distribution specific to each alternative (and hence no externality was assumed), thereby ensuring there would be no direct social competition over the monetary reward (Giraldeau and Caraco, 2000) nor normative pressure towards majority alignment (Cialdini and Goldstein, 2004; Mahmoodi et al., 2018). The value of social information was assumed to be only informational (Efferson et al., 2008; Nakahashi, 2007). Nevertheless, our model may apply to the context of normative social influences, because what we assumed here was modification in individual choice probabilities by social influences, irrespective of underlying motivations of conformity.

To model a compromise between individual trial-and-error learning and the frequency-based copying process, we formulated the social influences on reinforcement learning as a weighted average between the asocial (A) and social (S) processes of decision making, that is, $P_{i,t}=\left(1-\sigma \right)\,A_{i,t}+\sigma \,S_{i,t}$, where $P_{i,t}$ is the individual net probability of choosing an option i∈{r,s} at time t and σ is a weight given to the social influence (*copying weight*).

In addition, the level of social frequency dependence was determined by another social learning parameter θ (*conformity exponent*), such that $S_{i,t}=N_{i,t}^{\theta }/\left(N_{r,t}^{\theta }+N_{s,t}^{\theta }\right)$, where $N_{i}$ is the number of agents who chose option i (see the Materials and methods for the accurate formulation). The larger the θ, the more the net choice probability favours a common alternative chosen by the majority of a group at the moment (a conformity bias; Boyd and Richerson, 1985). Note that there is no actual social influence when θ=0 because in this case the ‘social influence’ favours a uniformly random choice, irrespective of whether it is a common behaviour.

Our model is a natural extension of both the asocial reinforcement learning and the model of ‘extreme conformity’ assumed in some previous models (e.g. Denrell and Le Mens, 2017), as these conditions can be expressed as a special case of parameter combinations. We explore the implications of this extension in the Discussion. The descriptions of the parameters are summarised in Table 1.

**Table 1.** Summary of the learning model parameters.

Symbol	Meaning	Range of the value
α	Learning rate	[0, 1]
β	Inverse temperature	[0, +∞]
α(1+β)	Susceptibility to the hot stove effect	
σ	Copying weight	[0, 1]
θ	Conformity exponent	[-∞, +∞]

### The collective behavioural rescue effect

Varying these two social learning parameters, σ and θ, systematically, we observed a mitigation of suboptimal risk aversion under positive frequency-based social influences. As shown in Figure 1c, even with a strong conformity bias (θ>1), social influence widened the region of parameter combinations where the majority of decision makers could escape from suboptimal risk aversion (the increase of the red area in Figure 1c). The increment of the area of adaptive risk seeking was greater with θ=1 than with θ=4. When θ=1, a large copying weight (σ) could eliminate almost all the area of risk aversion (Figure 1c; see also Figure 1—figure supplement 1 for a greater range of parameter combinations), whereas when θ=4, there was also a region in which optimal risk seeking was weakened (Figure 1d). On the other hand, such substantial switching of the majority to being risk seeking did not emerge in the negative risk premium (negative RP) task (Figure 1—figure supplement 3), although there was a parameter region where the proportion of suboptimal risk seeking relatively increased compared to that of individual learners (Figure 1—figure supplement 6). Naturally, increasing the copying weight σ→1 eventually approximated the chance-level performance in both positive and negative RP cases (Figure 1—figure supplement 1, Figure 1—figure supplement 3). In sum, simulations suggest that conformist social influence widely promoted risk seeking under the positive RP, and that such a promotion of risk seeking was less evident in the negative RP task.

Figure 2 highlights the extent to which risk aversion was relaxed through social influences. Individuals with positive σ>0 could maintain a high proportion of risk seeking even in the region of high susceptibility to the hot stove effect (α(β+1)>2). Although social learners eventually fell into a risk-averse regime with increasing α⁢(β+1), risk aversion was largely mitigated compared to the performance of individual learners who had σ=0. Interestingly, the probability of choosing the optimal risky option was maximised at an intermediate value of α⁢(β+1) when the conformity exponent was large θ=4 and the copying weight was high σ=0.5.

In the region of less susceptibility to the hot stove effect (α(β+1)<2), social influence could enhance individual optimal risk seeking up to the theoretical benchmark expected in individual reinforcement learning with an infinite time horizon (the solid curves in Figure 2). A socially induced increase in risk seeking in the region α(β+1)<2 was more evident with larger β, and hence with smaller α to satisfy α(β+1)<2. The smaller the learning rate α, the longer it would take to achieve the asymptotic equilibrium state, due to slow value updating. Asocial learners, as well as social learners with high σ (=0.5) coupled with high θ (=4), were still far from the analytical benchmark, whereas social learners with weak social influence σ=0.25 were nearly able to converge on the benchmark performance, suggesting that social learning might affect the speed of learning. Indeed, a longer time horizon T=1075 reduced the advantage of weak social learners in this α(β+1)<2 region because slow learners could now achieve the benchmark accuracy (Figure 2—figure supplement 1 and Figure 2—figure supplement 2).

Approaching the benchmark with an elongated time horizon, and the concomitant reduction in the advantage of social learners, was also found in the high susceptibility region α⁢(β+1)≫2 especially for those who had a high conformity exponent θ=4 (Figure 2—figure supplement 1). Notably, however, facilitation of optimal risk seeking became further evident in the other intermediate region 2<α(β+1)<4. This suggests that merely speeding up or slowing down learning could not satisfactorily account for the qualitative ‘choice shift’ emerging through social influences.

We obtained similar results across different settings of the multi-armed bandit task, such as a skewed payoff distribution in which either large or small payoffs were randomly drawn from a Bernoulli process (March, 1996; Denrell, 2007, Figure 1—figure supplement 4) and increased option numbers (Figure 1—figure supplement 5). Further, the conclusion still held for an alternative model in which social influences modified the belief-updating process (the value-shaping model; Najar et al., 2020) rather than directly influencing the choice probability (the decision-biasing model) as assumed in the main text thus far (see Supplementary Methods; Figure 1—figure supplement 2). One could derive many other more complex social learning processes that may operate in reality; however, the comprehensive search of possible model space is beyond the current interest. Yet, decision biasing was found to fit better than value shaping with our behavioural experimental data (Figure 6—figure supplement 2), leading us to focus our analysis on the decision-biasing model.

### The robustness of individual heterogeneity

**Figure 3.**
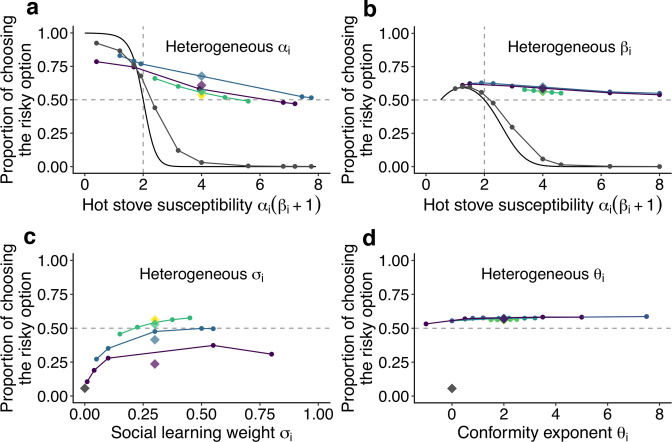
The effect of individual heterogeneity on the proportion of choosing the risky option in the two-armed bandit task. (**a**) The effect of heterogeneity of α, (**b**) β, (**c**) σ, and (**d**) θ. Individual values of a focal behavioural parameter were varied across individuals in a group of five. Other non-focal parameters were identical across individuals within a group. The basic parameter values assigned to non-focal parameters were α=0.5, β=7, σ=0.3, and θ=2, and groups’ mean values of the various focal parameters were matched to these basic values. We simulated 3 different heterogeneous compositions: The majority (3 of 5 individuals) potentially suffered the hot stove effect $\alpha_{i}(\beta_{i}+1)>2$ (**a, b**) or had the highest diversity in social learning parameters (c, d; purple); the majority were able to overcome the hot stove effect $\alpha_{i}(\beta_{i}+1)<2$ (**a, b**) or had moderate heterogeneity in the social learning parameters (c, d; blue); and all individuals had $\alpha_{i}(\beta_{i}+1)>2$ but smaller heterogeneity (green). The yellow diamond shows the homogeneous groups’ performance. Lines are drawn through average results across the same compositional groups. Each round dot represents a group member’s mean performance. The diamonds are the average performance of each group for each composition category. For comparison, asocial learners’ performance, with which the performance of social learners can be evaluated, is shown in gray. For heterogeneous α and β, the analytical solution of asocial learning performance is shown as a solid-line curve. We ran 20,000 replications for each group composition.

We have thus far assumed no parameter variations across individuals in a group to focus on the qualitative differences between social and asocial learners’ behaviour. However, individual differences in development, state, or experience or variations in behaviour caused by personality traits might either facilitate or undermine collective decision performance. Especially if a group is composed of both types of individuals, those who are less susceptible to the hot stove effect (α(β+1)<2) as well as those who are more susceptible α(β+1)>2, it remains unclear who benefits from the rescue effect: Is it only those individuals with α(β+1)>2 who enjoy the benefit, or can collective intelligence benefit a group as a whole? For the sake of simplicity, here we considered groups of five individuals, which were composed of either homogeneous (yellow in Figure 3) or heterogeneous (green, blue, purple in Figure 3) individuals. Individual values of a focal behavioural parameter were varied across individuals in a group. Other non-focal parameters were identical across individuals within a group. The basic parameter values assigned to non-focal parameters were α=0.5, β=7, σ=0.3, and θ=2, which were chosen so that the homogeneous group could generate the collective rescue effect. The groups’ mean values of the various focal parameters were matched to these basic values.

Figure 3a shows the effect of heterogeneity in the learning rate (α). Heterogeneous groups performed better on average than a homogeneous group (represented by the yellow diamond). The heterogeneous groups owed this overall improvement to the large rescue effect operating for individuals who had a high susceptibility to the hot stove effect (α⁢(β+1)≫2). On the other hand, the performance of less susceptible individuals (α(β+1)<2) was slightly undermined compared to the asocial benchmark performance shown in grey. Notably, however, how large the detrimental effect was for the low-susceptibility individuals depended on the group’s composition: The undermining effect was largely mitigated when low-susceptibility individuals (α(β+1)<2) made up a majority of a group (3 of 5; the blue line), whereas they performed worse than the asocial benchmark when the majority were those with high susceptibility (purple).

The advantage of a heterogeneous group was also found for the inverse temperature (β), although the impact of the group’s heterogeneity was much smaller than that for α (Figure 3b). Interestingly, no detrimental effect for individuals with α(β+1)<2 was found in association with the β variations.

On the other hand, individual variations in the copying weight (σ) had an overall detrimental effect on collective performance, although individuals in the highest diversity group could still perform better than the asocial learners (Figure 3c). Individuals who had an intermediate level of σ achieved relatively higher performance within the group than those who had either higher or lower σ. This was because individuals with lower σ could benefit less from social information, while those with higher σ relied so heavily on social frequency information that behaviour was barely informed by individual learning, resulting in maladaptive herding or collective illusion (Denrell and Le Mens, 2017; Toyokawa et al., 2019). As a result, the average performance decreased with increasing diversity in σ.

Such a substantial effect of individual differences was not observed in the conformity exponent θ (Figure 3d), where individual performance was almost stable regardless of whether the individual was heavily conformist ($\theta_{i}=8$) or even negatively dependent on social information ($\theta_{i}=-1$). The existence of a few conformists in a group could not itself trigger positive feedback among the group unless other individuals also relied on social information in a conformist-biased way, because the flexible behaviour of non-conformists could keep the group’s distribution nearly flat (i.e. $N_{s}\approx N_{r}$). Therefore, the existence of individuals with small θ in a heterogeneous group could prevent the strong positive feedback from being immediately elicited, compensating for the potential detrimental effect of maladaptive herding by strong conformists.

Overall, the relaxation of, and possibly the complete rescue from, a suboptimal risk aversion in repeated risky decision making emerged in a range of conditions in collective learning. It was not likely a mere speeding up or slowing down of learning process (Figure 2—figure supplement 1 and Figure 2—figure supplement 2), nor just an averaging process mixing performances of both risk seekers and risk-averse individuals (Figure 3). It depended neither on specific characteristics of social learning models (Figure 1—figure supplement 2) nor on the profile of the bandit task’s setups (Figure 1—figure supplement 4). Instead, our simulation suggests that self-organisation may play a key role in this emergent phenomenon. To seek a general mechanism underlying the observed collective behavioural rescue, in the next section we show a reduced, approximated differential equation model that can provide qualitative insights into the collective decision-making dynamics observed above.

### The simplified population dynamics model

To obtain a qualitative understanding of self-organisation that seems responsible for the pattern of adaptive behavioural shift observed in our individual-based simulation, we made a reduced model that approximates temporal changes of behaviour of an ‘average’ individual, or in other words, average dynamics of a population of multiple individuals, where the computational details of reinforcement learning were purposely ignored. Such a dynamic modelling approach has been commonly used in population ecology and collective animal behaviour research and has proven highly useful in disentangling the factors underlying complex systems (e.g. Beckers et al., 1990; Goss et al., 1989; Seeley et al., 1991; Sumpter and Pratt, 2003; Harrison et al., 2001).

**Figure 4.**
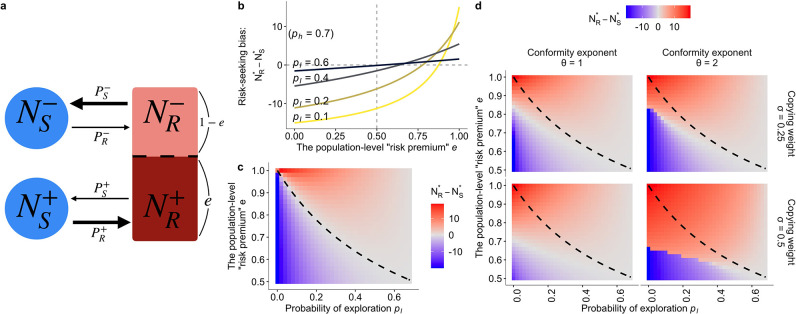
The population dynamics model. (**a**) A schematic diagram of the dynamics. Solid arrows represent a change in population density between connected states at a time step. The thicker the arrow, the larger the per-capita rate of behavioural change. (**b, c**) The results of the asocial, baseline model where $P_{S}^{-}=P_{R}^{+}=p_{h}$ and $P_{R}^{-}=P_{S}^{+}=p_{l}$ ($p_{h}>p_{l}$). Both figures show the equilibrium bias towards risk seeking (i.e., $N_{r}^{⋆}-N_{s}^{⋆}$) as a function of the degree of risk premium e as well as of the per-capita probability of moving to the less preferred behavioural option $p_{l}$. (**b**) The explicit form of the curve is given by $-n\,\left(p_{h}-p_{l}\right)\,\left\{\left(1-e\right)\,p_{h}-e\,p_{l}\right\}/\left(p_{h}+p_{l}\right)\,\left\{\left(1-e\right)\,p_{h}+e\,p_{l}\right\}$. (**c**) The dashed curve is the analytically derived neutral equilibrium of the asocial system that results in $N_{R}^{*}=N_{S}^{*}$, given by $e=p_{h}/\left(p_{h}+p_{l}\right)$. (**d**) The equilibrium of the collective behavioural dynamics with social influences. The numerical results were obtained with $N_{S,t=0}^{-}=N_{S,t=0}^{+}=5$, $N_{R,t=0}=10$, and $p_{h}=0.7$.

Specifically, we considered a differential equation that focuses only on increases and decreases in the number of individuals who are choosing the risky option ($N_{R}$) and the safe option ($N_{S}$) with either a positive (+) or a negative (-) ‘attitude’ (or preference) towards the risky option (Figure 4a). The part of the population that has a positive attitude ($N_{S}^{+}$ and $N_{R}^{+}$) is more likely to move on to, and stay at, the risky option, whereas the other part of the population that has a negative attitude ($N_{S}^{-}$ and $N_{R}^{-}$) is more likely to move on to, and stay at, the safe option. Note that movements in the opposite direction also exist, such as moving on to the risky option when having a negative attitude ($P_{R}^{-}$), but at a lower rate than $P_{S}^{-}$, depicted by the thickness of the arrows in Figure 4a. We defined that the probability of moving towards an option matched with their attitude ($P_{S}^{-}=P_{R}^{+}=p_{h}$) was higher than that of moving in the opposite direction ($P_{R}^{-}=P_{S}^{+}=p_{l}$), that is, $p_{h}>p_{l}$. The probability $p_{l}$ and $p_{h}$ can be seen approximately as the per capita rate of exploration and exploitation, respectively.

**Figure 4—figure supplement 1.**
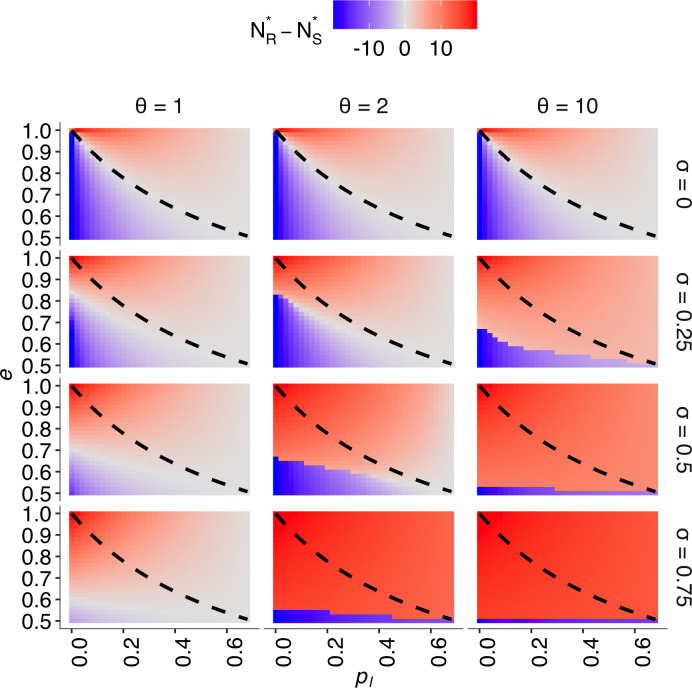
The result of the differential equation model. The effect of both the per capita probability of exploration $p_{l}$ and e (i.e. the ratio of individuals who prefer behavioural state R) on the equilibrium degree of risk seeking (i.e. $N_{R}^{*}-N_{S}^{*}$), across the different combinations of social influence parameters. Different social influence weights are shown from top to bottom (σ∈{0,0.25,0.5,0.75}). Different conformity exponents are shown from left to right (θ∈{1,2,10}). The dashed curve is $e=p_{h}/\left(p_{h}+p_{l}\right)$. The numeric solution was obtained with conditions $N_{S,t=0}^{-}=N_{S,t=0}^{+}=5$, $N_{R,t=0}=10$, and $p_{h}=0.7$.

An attitude can change when the risky option is chosen. We assumed that a proportion e (0≤e≤1) of the risk-taking part of the population would have a good experience, thereby holding a positive attitude (i.e. $N_{R}^{+}=e\,N_{R}$). On the other hand, the rest of the risk-taking population would have a negative attitude (i.e. $N_{R}^{-}=\left(1-e\right)\,N_{R}$). This proportion e can be interpreted as an approximation of the risk premium under the Gaussian noise of risk, because the larger e is, the more individuals one would expect would encounter a better experience than when making the safe choice. The full details are shown in the Materials and methods (Table 2).

**Table 2.** Summary of the differential equation model parameters.

Symbol	Meaning	Range of the value
$N_{R}^{+}$	Density of individuals choosing R and preferring R	$N_{R}^{+}=e\,N_{R}$
$N_{R}^{-}$	Density of individuals choosing R and preferring S	$N_{R}^{-}=\left(1-e\right)\,N_{R}$
$N_{S}^{+}$	Density of individuals choosing S and preferring R	
$N_{S}^{-}$	Density of individuals choosing S and preferring S	
$p_{l}$	Per capita rate of moving to the unfavourable option	$0\leq p_{l}\leq p_{h}\leq 1$
$p_{h}$	Per capita rate of moving to the favourable option	$0\leq p_{l}\leq p_{h}\leq 1$
e	Per capita rate of becoming enchanted with the risky option	[0,1]
σ	Social influence weight	[0,1]
θ	Conformity exponent	[-∞,+∞]

To confirm that this approximated model can successfully replicate the fundamental property of the hot stove effect, we first describe the asocial behavioural model without social influence. The baseline, asocial dynamic system has a locally stable non-trivial equilibrium that gives $N_{S}^{⋆}\geq 0$ and $N_{R}^{⋆}\geq 0$, where $N^{⋆}$ means the equilibrium density at which the system stops changing ($d\,N_{S}^{⋆}/d\,t=d\,N_{R}^{⋆}/d\,t=0$). At equilibrium, the ratio between the number of individuals choosing the safe option S and the number choosing the risky option R is given by $N_{S}^{⋆}:N_{R}^{⋆}=e\,\left(p_{l}/p_{h}\right)+\left(1-e\right)\,\left(p_{h}/p_{l}\right):1$, indicating that risk aversion (defined as the case where a larger part of the population chooses the safe option; $N_{S}^{⋆}>N_{R}^{⋆}$) emerges when the inequality $e<P_{S}^{-}/(P_{S}^{-}+P_{R}^{-})=p_{h}/(p_{h}+p_{l})$ holds.

Figure 4b visually shows that the population is indeed attracted to the safe option S (that is, $N_{S}^{⋆}>N_{R}^{⋆}$) in a wide range of the parameter region even when there is a positive ‘risk premium’ defined as e>1/2. Although individuals choosing the risky option are more likely to become enchanted with the risky option than to be disappointed (i.e., $eN_{R}=N_{R}^{+}>(1-e)N_{R}=N_{R}^{-}$), the risk-seeking equilibrium (defined as $N_{S}^{⋆}<N_{R}^{⋆}$) becomes less likely to emerge as the exploration rate $p_{l}$ decreases, consistent with the hot stove effect caused by asymmetric adaptive sampling (Denrell, 2007). Risk seeking never emerges when e≤1/2, which is also consistent with the results of reinforcement learning.

This dynamics model provides an illustrative understanding of how the asymmetry of adaptive sampling causes the hot stove effect. Consider the case of high inequality between exploitation ($p_{h}$) and exploration ($p_{l}$), namely, $p_{h}\gg p_{l}$. Under such a condition, the state $S^{-}$, that is choosing the safe option with the negative inner attitude –, becomes a ‘dead end’ from which individuals can seldom escape once entered. However, if the inequality $p_{h}\geq p_{l}$ is not so large that a substantial fraction of the population now comes back to $R^{-}$ from $S^{-}$, the increasing number of people belonging to $R^{+}$ (that is, $N_{R}^{+}$) could eventually exceed the number of people ‘spilling out’ to $S^{-}$. Such an illustrative analysis shows that the hot stove effect can be overcome if the number of people who get stuck in the dead end $S^{-}$ can somehow be reduced. And this is possible if one can increase the ‘come-backs’ to $R^{-}$. In other words, if any mechanisms can increase $P_{R}^{-}$ in relation to $P_{S}^{-}$, the hot stove effect should be overcome.

Next, we assumed a frequency-dependent reliance on social information operating in this population dynamics. Specifically, we considered that the net per capita probability of choosing each option, P, is composed of a weighted average between the asocial baseline probability (p) and the social frequency influence (F), namely, P=(1-σ)⁢p+σ⁢F. Again, σ is the weight of social influence, and we also assumed that there would be the conformity exponent θ in the social frequency influence F such that $F=N_{i}^{\theta }/\left(N_{S}^{\theta }+N_{R}^{\theta }\right)$ where i∈{S,R} (see Materials and methods).

**Figure 5.**
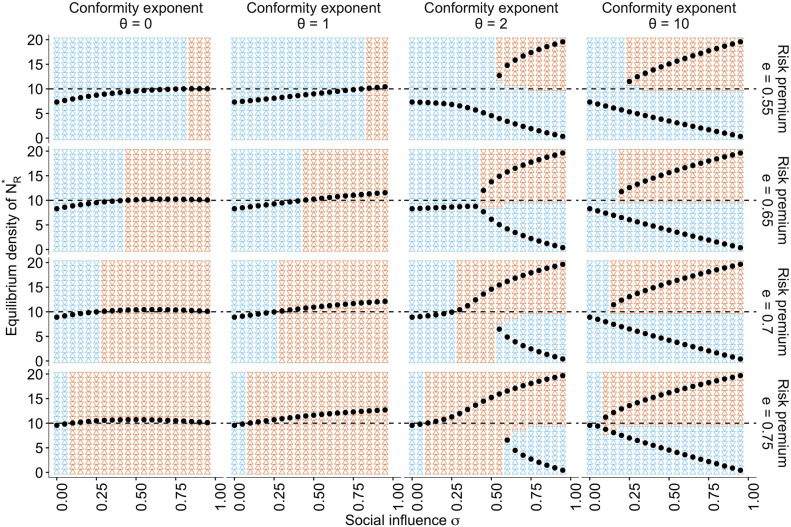
The approximate bifurcation analysis. The relationships between the social influence weight σ and the equilibrium number of individuals in the risky behavioural state $N_{R}^{⋆}$ across different conformity exponents θ∈{0,1,2,10} and different values of risk premium e∈{0.55,0.65,0.7,0.75}, are shown as black dots. The background colours indicate regions where the system approaches either risk aversion ($N_{R}^{⋆}<N_{S}^{⋆}$; blue) or risk seeking ($N_{R}^{⋆}>N_{S}^{⋆}$; red). The horizontal dashed line is $N_{R}=N_{S}=10$. Two locally stable equilibria emerge when θ≥2, which suggests that the system has a bifurcation when σ is sufficiently large. The other parameters are set to $p_{h}=0.7$, $p_{l}=0.2$, and N=20.

Through numerical analyses, we have confirmed that social influence can indeed increase the flow-back rate $P_{R}^{-}$, which raises the possibility of risk-seeking equilibrium $N_{R}^{⋆}>N_{S}^{⋆}$ (Figure 4d; see Figure 4—figure supplement 1 for a wider parameter region). For an approximation of the bifurcation analysis, we recorded the equilibrium density of the risky state $N_{R}^{⋆}$ starting from various initial population distributions (that is, varying $N_{R,t=0}$ and $N_{S,t=0}=20-N_{R,t=0}$). Figure 5 shows the conditions under which the system ends up in risk-seeking equilibrium. When the conformity exponent θ is not too large (θ<10), there is a region that risk seeking can be a unique equilibrium, irrespective of the initial distribution, and attracting the population even from an extremely biased initial distribution such as $N_{R,t=0}=0$ (Figure 5).

**Figure 5—figure supplement 1.**
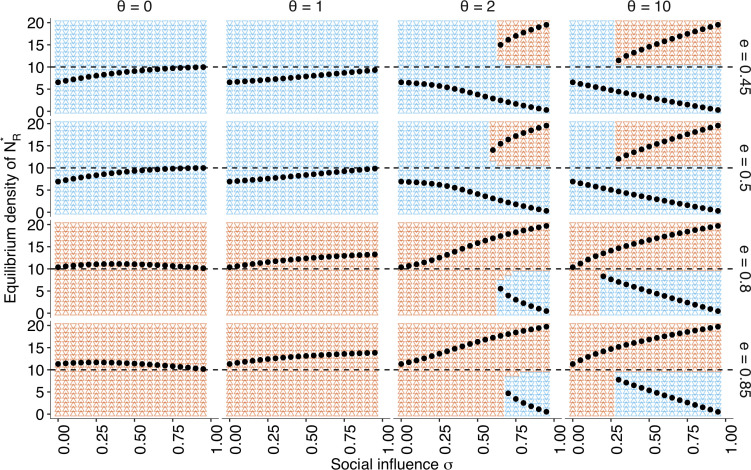
The approximate bifurcation analysis. The relationship between the social influence weight σ and the equilibrium number of individuals choosing the risky alternative $N_{R}^{⋆}$ across the different conformity exponents θ(∈{0,1,2,10}), shown as black dots. The triangular points shown in the background of each panel indicate regions in which the group approaches risk aversion (i.e., $N_{R}^{⋆}<10$; blue) or the risk-seeking equilibrium (i.e. $N_{R}^{⋆}>10$; red). Two different equilibria mean that the system has a bifurcation under a given σ. The direction of the background triangles indicates whether $N_{R}$ increases (Δ) or decreases (∇) relative to its starting position. The other parameters are set to $p_{h}=0.7$, $p_{l}=0.2$.

Under the conformist bias θ≥2, two locally stable equilibria exist. Strong positive feedback dominates the system when both σ and θ are large. Therefore, the system can end up in either of the equilibria depending solely on the initial density distribution, consistent with the conventional view of herding (Denrell and Le Mens, 2017; Toyokawa et al., 2019). This is also consistent with a well-known result of collective foraging by pheromone trail ants, which react to social information in a conformity-like manner (Beckers et al., 1990; Harrison et al., 2001).

Notably, however, even with a positive conformist bias, such as θ=2, there is a region with a moderate value of σ where risk seeking remains a unique equilibrium when the risk premium was high (e≥0.7). In this regime, the benefit of collective behavioural rescue can dominate without any possibility of maladaptive herding.

It is worth noting that in the case of θ=0, where individuals make merely a random choice at a rate σ, risk aversion is also relaxed (Figure 5, the leftmost column), and the adaptive risky shift even emerges around 0.25<σ<1. However, this ostensible behavioural rescue is due solely to the pure effect of additional random exploration that reduces $P_{S}^{-}/\left(P_{S}^{-}+P_{R}^{-}\right)$, mitigating stickiness to the dead-end status $S^{-}$. When σ→1 with θ=0, therefore, the risky shift eventually disappears because the individuals choose between S and R almost randomly.

However, the collective risky shift observed in the conditions of θ>0 cannot be explained solely by the mere addition of exploration. A weak conformist bias (i.e. a linear response to the social frequency; θ=1) monotonically increases the equilibrium density $N_{R}^{⋆}$ with increasing social influence σ, which goes beyond the level of risky shift observed with the addition of random choice (Figure 5). Therefore, although the collective rescue might indeed owe its part of the mitigation of the hot stove effect to increasing exploration, the further enhancement of risk seeking cannot be fully explained by it alone.

The key is the interaction between negative and positive feedback. As we discussed above, risk aversion is reduced if the ratio $P_{S}^{-}/\left(P_{S}^{-}+P_{R}^{-}\right)$ decreases, either by increasing $P_{R}^{-}$ or reducing $P_{S}^{-}$. The per individual probability of choosing the safe option with the negative attitude, that is, $P_{S}^{-}=\left(1-\sigma \right)\,p_{h}+\sigma \,N_{S}^{\theta }/\left(N_{R}^{\theta }+N_{S}^{\theta }\right)$, becomes smaller than the baseline exploitation probability $p_{h}$, when $N_{S}^{\theta }/(N_{R}^{\theta }+N_{S}^{\theta })<p_{h}$. Even though the majority of the population may still choose the safe alternative and hence $N_{S}>N_{R}$, the inequality $N_{S}^{\theta }/(N_{R}^{\theta }+N_{S}^{\theta })<p_{h}$ can nevertheless hold if one takes a sufficiently small value of θ. Crucially, the reduction of $P_{S}^{-}$ leads to a further reduction of $P_{S}^{-}$ itself through decreasing $N_{S}^{-}$, thereby further decreasing the social influence supporting the safe option. Such a negative feedback process weakens the concomitant risk aversion. Naturally, this negative feedback is maximised with θ=0.

Once the negative feedback has weakened the underlying risk aversion, the majority of the population eventually choose the risky option, an effect evident in the case of θ=0 (Figure 5). What uniquely operates in cases of θ>0 is that because $N_{R}$ is a majority by now, positive feedback starts. Thanks to the conformist bias, the inequality $N_{R}>N_{S}$ is further *amplified*. In this phase, the larger θ, the stronger the concomitant relationship $N_{S}^{\theta }/\left(N_{R}^{\theta }+N_{S}^{\theta }\right)\ll p_{h}$. Such positive feedback will never operate with θ≤0.

In conclusion, it is the synergy of negative and positive feedback that explains the full range of adaptive risky shift. Neither positive nor negative feedback alone can account for both accuracy and flexibility emerging through collective learning and decision making. The results are qualitatively unchanged across a range of different combinations of e, $p_{l}$, and $p_{h}$ (Figure 4—figure supplement 1 and Figure 5—figure supplement 1). It is worth noting that when e<0.5, this social frequency-dependent population tends to exhibit risk aversion (Figure 5—figure supplement 1), consistent with the result of the agent-based simulation for the case where the mean payoff of the risky option was smaller than that of the safe option (Figure 1—figure supplement 3). Therefore, the system does not mindlessly prefer risk seeking, but it becomes risk prone only when to do so is favourable in the long run.

### An experimental demonstration

One hundred eighty-five adult human subjects performed the individual task without social interactions, while 400 subjects performed the task collectively with group sizes ranging from 2 to 8. We confirmed that the model predictions were qualitatively unchanged across the experimental settings used in the online experiments (Figure 1—figure supplement 5).

We used four different task settings. Three of them were positive risk premium (positive RP) tasks that had an optimal risky alternative, while the other was a negative risk premium (negative RP) task that had a suboptimal risky alternative. On the basis of both the agent-based simulation (Figure 1 and Figure 1—figure supplement 3) and the population dynamics (Figure 5 and Figure 5—figure supplement 1), we hypothesised that conformist social influence promotes risk seeking to a lesser extent when the RP is negative than when it is positive. We also expected that whether the collective rescue effect emerges under positive RP settings depends on learning parameters such as $\alpha_{i}(\beta_{i}+1)$ (Figure 1—figure supplement 5d-f).

The Bayesian model comparison (Stephan et al., 2009) revealed that participants in the group condition were more likely to employ decision-biasing social learning than either asocial reinforcement learning or the value-shaping process (Figure 6—figure supplement 2). Therefore, in the following analysis, we focus on results obtained from the decision-biasing model fit. Individual parameters were estimated using a hierarchical Bayesian method whose performance had been supported by the parameter recovery (Figure 6—figure supplement 3).

Parameter estimation (Table 3) showed that individuals in the group condition across all four tasks were likely to use social information in their decision making at a rate ranging between 4% and 18% (Mean σ; Table 3), and that mean posterior values of θ were above 1 for all four tasks. These suggest that participants were likely to use a mix of individual reinforcement learning and conformist social learning.

**Table 3.** Means and 95% Bayesian credible intervals (shown in square brackets) of the global parameters of the learning model. The group condition and individual condition are shown separately. All parameters satisfied the Gelman–Rubin criterion $\hat{R}<1.01$. All estimates are based on over 500 effective samples from the posterior.

Task category	Positive risk premium (positive RP)			Negative risk premium (negative RP)
Task	1-risky-1-safe	1-risky-3-safe	2-risky-2-safe	1-risky-1-safe
Group	n = 123	n = 97	n = 87	n = 93
μ_logitα_	–2.2 [-2.8,–1.5]	–1.8 [-2.3,–1.4]	–1.7 [-2.1,–1.3]	–0.09 [-0.7, 0.6]
(Mean α)	0.10 [0.06, 0.18]	0.14 [0.09, 0.20]	0.15 [0.11, 0.21]	0.48 [0.3, 0.6]
μ_logitβ_	1.4 [1.1, 1.6]	1.5 [1.3, 1.8]	1.3 [1.0, 1.5]	1.2 [1.0, 1.5]
(Mean β)	4.1 [3.0, 5.0]	4.5 [3.7, 6.0]	3.7 [2.7, 4.5]	3.3 [2.7, 4.5]
μ_logitα_	–2.4 [-3.1,–1.8]	–2.1 [-2.6,–1.6]	–2.1 [-2.5,–1.7]	–2.0 [-2.7,–1.5]
(Mean σ)	0.08 [0.04, 0.14]	0.11 [0.07, 0.17]	0.11 [0.08, 0.15]	0.12 [0.06. 0.18]
μ_θ_ = mean θ	1.4 [0.58, 2.3]	1.6 [0.9, 2.4]	1.8 [1.0, 2.9]	1.6 [0.9, 2.3]
Individual	n = 45	n = 51	n = 64	n = 25
μ_logitα_	–2.1 [-3.1,–0.87]	–2.1 [-2.6,–1.6]	–1.3 [-2.1,–0.50]	–1.3 [-2.2,–0.4]
(Mean α)	0.11 [0.04, 0.30]	0.11 [0.07, 0.17]	0.21 [0.11, 0.38]	0.2 [0.1, 0.4]
μ_logitβ_	0.42 [-0.43, 1.1]	0.91 [0.63, 1.2]	0.76 [0.42, 1.1]	1.2 [0.9, 1.4]
(Mean β)	1.5 [0.65, 3.0]	2.5 [1.9, 3.3]	2.1 [1.5, 3.0]	3.3 [2.5, 4.1]

To address whether the behavioural data are well explained by our social learning model and whether collective rescue was indeed observed for social learning individuals, we conducted agent-based simulations of the fit computational model with the calibrated parameters, including 100,000 independent runs for each task setup (see Materials and methods).

**Figure 6.**
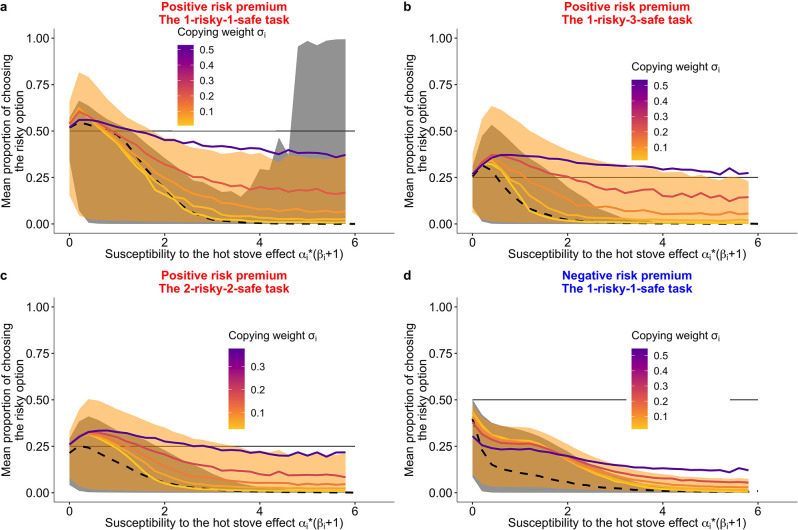
Prediction of the fit learning model. Results of a series of agent-based simulations with individual parameters that were drawn randomly from the best fit global parameters. Independent simulations were conducted 100,000 times for each condition. Group size was fixed to six for the group condition. Lines are means (black-dashed: individual, coloured-solid: group) and the shaded areas are 80% Bayesian credible intervals. Mean performances of agents with different $\sigma_{i}$ are shown in the colour gradient. (**a**) A two-armed bandit task. (**b**) A 1-risky-3-safe (four-armed) bandit task. (**c**) A 2-risky-2-safe (four-armed) bandit task. (**d**) A negative risk premium two-armed bandit task.

The results of the agent-based simulations agreed with our hypotheses (Figure 6). Overall, the 80% Bayesian credible intervals of the predicted performance of the group condition (shades of orange in Figure 6) cover an area of more risk taking than the area covered by the individual condition (shades of grey). As predicted, in the negative RP task, social learning promoted suboptimal risk taking for some values of α⁢(β+1), but the magnitude looked smaller compared to in the positive RP tasks. Additionally, increasing $\sigma_{i}$ led to an increasing probability of risk taking in the positive RP tasks (Figure 6a–c), whereas in the negative RP task, increasing σ did not always increase risk taking (Figure 6d).

**Figure 6—figure supplement 1.**
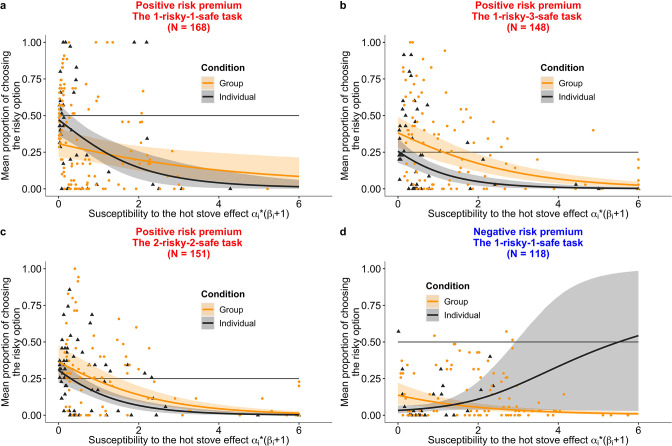
Experimental results with the mixed logit model regression. The black triangles are subjects in the individual learning condition; the orange dots are those in the group condition with group sizes ranging from 2 to 8. The solid lines are predictions from a mixed logit model for the individual condition (black) and for the group condition (orange), with the shaded area showing the 95% Bayesian credible intervals (CIs). (**a**) A two-armed bandit task (N=168). (**b**) A 1-risky-3-safe (four-armed) bandit task (N=148). (**c**) A 2-risky-2-safe (four-armed) bandit task (N=151). (**d**) A negative risk premium (RP) two-armed bandit task (N=118). The width of the CI for the individual condition in the negative RP task is due to the lack of data points in the region. The *x* axis is $\alpha_{i}\,\left(\beta_{i}+1\right)$, namely, the susceptibility to the hot stove effect. (**a**, **b,** and **d**) The *y* axis is the mean proportion of choosing the risky alternative averaged over the second half of the trials. (**c**) The *y* axis is the mean proportion of choosing the optimal risky alternative averaged over the second half of the trials. The horizontal lines show the chance-level probability.

**Figure 6—figure supplement 2.**
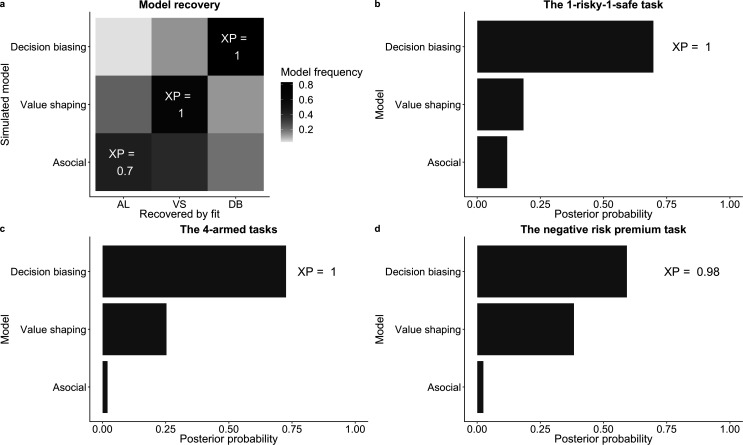
Bayesian model comparison. (**a**) The model recovery performance: model frequencies (dark shade) and exceedance probability (XP) for each pair of simulated and fitted models, calculated by the Widely Applicable Information Criterion (WAIC). (b–d) Model comparison results. The lengths of the bars indicate model frequencies. Exceedance probability (XP) of the decision-biasing model is shown.

**Figure 6—figure supplement 3.**
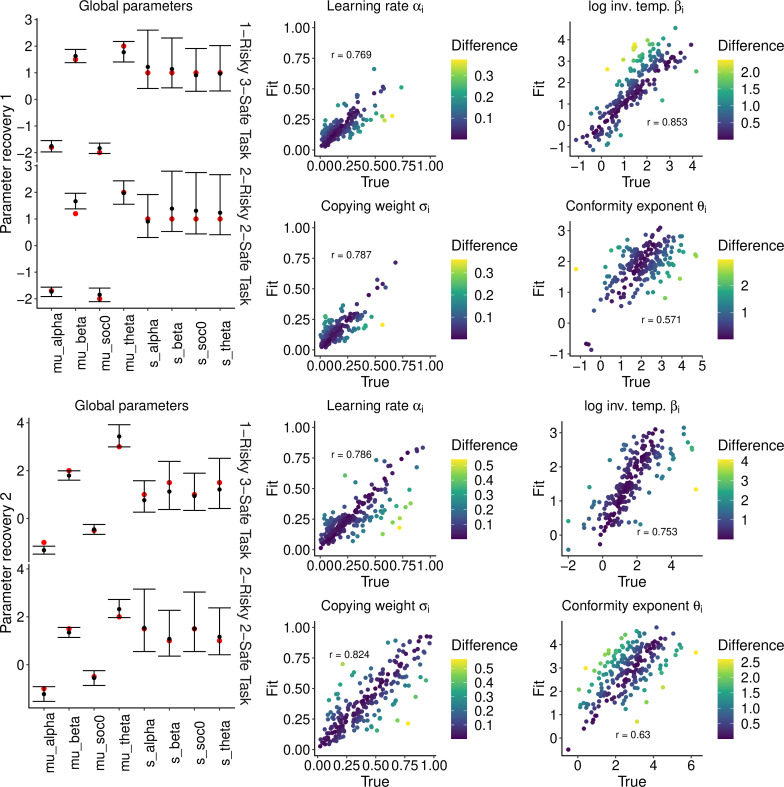
The parameter recovery performance. The top half and bottom half of the figure are the results of parameter recovery test 1 and 2, respectively. The left column shows the global parameters fitted for each of the two four-armed bandit tasks, the 1-risky-3-safe task (N=105) and the 2-risky-2-safe task (N=105). The red points are the true values and the black points are the mean posterior values (i.e. recovered values). The 95% Bayesian credible intervals are shown with error bars. The middle and right column are individual-level parameters across the two task conditions (N=210). The *x* axis is the true value and the *y* axis is the fitted (i.e. the mean posterior) individual value. The differences between the true value and the estimated value are shown in different colours (Dark: fit well). The Pearson’s correlation coefficients between the true and fitted values are shown.

However, a complete switch of the majority’s behaviour from the suboptimal safe options to the optimal risky option (i.e. $P_{r}>0.5$ for the two-armed task and $P_{r}>0.25$ for the four-armed task) was not widely observed. This might be because of the low copying weight (σ), coupled with the lower $\alpha_{i}\,\left(\beta_{i}+1\right)$ of individual learners (mean [median] = 0.8 [0.3]) than that of social learners (mean [median] = 1.1 [0.5]; Table 3). The weak average reliance on social learning ($\sigma_{i}$) hindered the strong collective rescue effect because strong positive feedback was not robustly formed.

To quantify the effect size of the relationship between the proportion of risk taking and each subject’s best fit learning parameters, we analysed a generalised linear mixed model (GLMM) fitted with the experimental data (see Materials and methods; Table 4). Within the group condition, the GLMM analysis showed a positive effect of $\sigma_{i}$ on risk taking for every task condition (Table 4), which supports the simulated pattern. Also consistent with the simulations, in the positive RP tasks, subjects exhibited risk aversion more strongly when they had a higher value of $\alpha_{i}(\beta_{i}+1)$ (Figure 6—figure supplement 1a-c). There was no such clear trend in data from the negative RP task, although we cannot make a strong inference because of the large width of the Bayesian credible interval (Figure 6—figure supplement 1d). In the negative RP task, subjects were biased more towards the (favourable) safe option than subjects in the positive RP tasks (i.e. the intercept of the GLMM was lower in the negative RP task than in the others).Table 2.

**Table 4.** Means and 95% Bayesian credible intervals (CIs; shown in square brackets) of the posterior estimations of the mixed logit model (generalised linear mixed model) that predicts the probability of choosing the risky alternative in the second half of the trial (t>35). All parameters satisfied the Gelman–Rubin criterion $\hat{R}<1.01$. All estimates are based on over 500 effective samples from the posterior. Coefficients whose CI is either below or above 0 are highlighted.

Task category	Positive Risk Premium (positive RP)			Negative Risk Premium (negative RP)
Task	1-risky-1-safe	1-risky-3-safe	2-risky-2-safe	1-risky-1-safe
	n = 168	n = 148	n = 151	n = 118
Intercept	–0.1 [-0.6, 0.3]	–1.1 [-1.5,–0.6]	–0.8 [-1.2,–0.4]	–3.5 [-4.4,–2.7]
Susceptibility to the hot stove effect (α(β+1))	–0.9 [-1.3,–0.4]	–1.0 [-1.5,–0.5]	–0.9 [-1.3,–0.6]	0.6 [-0.1, 1.4]
Group (no = 0/yes = 1)	0.0 [-0.7, 0.7]	–0.2 [-1.0, 0.7]	0.4 [-0.5, 1.2]	3.8 [2.7, 4.9]
Group × α(β+1)	0.6 [0.0, 1.1]	0.4 [0.0, 0.9]	0.3 [-0.1, 0.7]	–1.1 [-1.9,–0.3]
Group × copying weight σ	1.4 [0.5, 2.3]	1.9 [0.8, 3.0]	2.2 [0.4, 4.0]	3.8 [2.2, 5.3]
Group × conformity exponent θ	–0.7 [-0.9,–0.5]	0.2 [0.0, 0.5]	–0.3 [-0.5,–0.1]	–1.8 [-2.1,–1.5]

In sum, the experimental data analysis supports our prediction that conformist social influence promotes favourable risk taking even if individuals are biased towards risk aversion. The GLMM generally agreed with the theoretical prediction, and the fitted computational model that was supported by the Bayesian model comparison confirmed that the observed pattern was indeed likely to be a product of the collective rescue effect by conformist social learning. As predicted, the key was the balance between individual learning and the use of social information. In the Discussion, we consider the effect of the experimental setting on human learning strategies, which can be explored in future studies.

## Discussion

We have demonstrated that frequency-based copying, one of the most common forms of social learning strategy, can rescue decision makers from committing to adverse risk aversion in a risky trial-and-error learning task, even though a majority of individuals are potentially biased towards suboptimal risk aversion. Although an extremely strong reliance on conformist influence can raise the possibility of getting stuck on a suboptimal option, consistent with the previous view of herding by conformity (Raafat et al., 2009; Denrell and Le Mens, 2017), the mitigation of risk aversion and the concomitant collective behavioural rescue could emerge in a wide range of situations under modest use of conformist social learning.

Neither the averaging process of diverse individual inputs nor the speeding up of learning could account for the rescue effect. The individual diversity in the learning rate ($\alpha_{i}$) was beneficial for the group performance, whereas that in the social learning weight ($\sigma_{i}$) undermines the average decision performance, which could not be explained simply by a monotonic relationship between diversity and wisdom of crowds (Lorenz et al., 2011). Self-organisation through collective behavioural dynamics emerging from the experience-based decision making must be responsible for the seemingly counter-intuitive phenomenon of collective rescue.

Our simplified differential equation model has identified a key mechanism of the collective behavioural rescue: the synergy of positive and negative feedback. Despite conformity, the probability of choosing the suboptimal option can decrease from what is expected by individual learning alone. Indeed, an inherent individual preference for the safe alternative, expressed by the softmax function $e^{\beta Q_{s}}/(e^{\beta Q_{s}}+e^{\beta Q_{r}})$, is mitigated by the conformist influence $N_{s}^{\theta }/(N_{s}^{\theta }+N_{r}^{\theta })$ as long as the former is larger than the latter. In other words, risk-aversion was mitigated not because the majority chose the risky option, nor were individuals simply attracted towards the majority. Rather, participants’ choices became risker even though the majority chose the safer alternative at the outset. Under social influences (either because of informational or normative motivations), individuals become more explorative, likely to continue sampling the risky option even after he/she gets disappointed by poor rewards. Once individual risk aversion is reduced, there will exist fewer individuals choosing the suboptimal safe option, which further reduces the number of majority choosing the safe option. This negative feedback facilitates individuals revisiting the risky alternative. Such an attraction to the risky option allows more individuals, including those who are currently sceptical about the value of the risky option, to experience a large bonanza from the risky option, which results in ‘gluing’ them to the risky alternative for a while. Once a majority of individuals get glued to the risky alternative, positive feedback from conformity kicks in, and optimal risk seeking is further strengthened.

Models of conformist social influences have suggested that influences from the majority on individual decision making can lead a group as a whole to collective illusion that individuals learn to prefer any behavioural alternatives supported by many other individuals (Denrell and Le Mens, 2007; Denrell and Le Mens, 2017). However, previous empirical studies have repeatedly demonstrated that collective decision making under frequency-based social influences is broadly beneficial and can maintain more flexibility than what suggested by models of herding and collective illusion (Toyokawa et al., 2019; Aplin et al., 2017; Beckers et al., 1990; Seeley et al., 1991; Harrison et al., 2001; Kandler and Laland, 2013). For example, Aplin et al., 2017 demonstrated that populations of great tits (*Parus major*) could switch their behavioural tradition after an environmental change even though individual birds were likely to have a strong conformist tendency. A similar phenomenon was also reported in humans (Toyokawa et al., 2019).

Although these studies did not focus on risky decision making, and hence individuals were not inherently biased, experimentally induced environmental change was able to create such a situation where a majority of individuals exhibited an out-dated, suboptimal behaviour. However, as we have shown, a collective learning system could rescue their performance even though the individual distribution was strongly biased towards the suboptimal direction at the outset. The great tit and human groups were able to switch their tradition because of, rather than despite, the conformist social influence, thanks to the synergy of negative and positive feedback processes. Such the synergistic interaction between positive and negative feedback could not be predicted by the collective illusion models where individual decision making is determined fully by the majority influence because no negative feedback would be able to operate.

Through online behavioural experiments using a risky multi-armed bandit task, we have confirmed our theoretical prediction that simple frequency-based copying could mitigate risk aversion that many individual learners, especially those who had higher learning rates or lower exploration rates or both, would have exhibited as a result of the hot stove effect. The mitigation of risk aversion was also observed in the negative RP task, in which social learning slightly undermined the decision performance. However, because riskiness and expected reward are often positively correlated in a wide range of decision-making environments in the real world (Frank, 2009; Pleskac and Hertwig, 2014), the detrimental effect of reducing optimal risk aversion when risk premium is negative could be negligible in many ecological circumstances, making the conformist social learning beneficial in most cases.

Yet, a majority, albeit a smaller one, still showed risk aversion. The weak reliance on social learning, which affected less than 20% of decisions, was unable to facilitate strong positive feedback. The little use of social information might have been due to the lack of normative motivations for conformity and to the stationarity of the task. In a stable environment, learners could eventually gather enough information as trials proceeded, which might have made them less curious about information gathering including social learning (Rendell et al., 2010). In reality, people might use more sophisticated social learning strategies whereby they change the reliance on social information flexibly over trials (Deffner et al., 2020; Toyokawa et al., 2017; Toyokawa et al., 2019). Future research should consider more strategic use of social information, and will look at the conditions that elicit heavier reliance on the conformist social learning in humans, such as normative pressures for aligning with majority, volatility in the environment, time pressure, or an increasing number of behavioural options (Muthukrishna et al., 2016), coupled with much larger group sizes (Toyokawa et al., 2019).

The low learning rate α, which was at most 0.2 for many individuals in all the experimental task except for the negative RP task, should also have hindered the potential benefits of collective rescue in our current experiment, because the benefit of mitigating the hot stove effect would be minimal or hardly realised under such a small susceptibility to the hot stove effect. Although we believe that the simplest stationary environment was a necessary first step in building our understanding of the collective behavioural rescue effect, we would suggest that future studies use a temporally unstable (‘restless’) bandit task to elicit both a higher learning rate and a heavier reliance on social learning, so as to investigate the possibilities of a stronger effect. Indeed, previous studies with changing environments have reported a learning rate as high as α>0.5 (Toyokawa et al., 2017; Toyokawa et al., 2019; Deffner et al., 2020), under which individual learners should have suffered the hot stove trap more often.

Information about others’ payoffs might also be available in addition to inadvertent social frequency cues in some social contexts (Bault et al., 2011; Bolton and Harris, 1999). Knowing others’ payoffs allows one to use the ‘copy-successful-individuals’ strategy, which has been suggested to promote risk seeking irrespective of the risk premium because at least a subset of a population can be highly successful by sheer luck in risk taking (Baldini, 2012; Baldini, 2013; Takahashi and Ihara, 2019). Additionally, cooperative communications may further amplify the suboptimal decision bias if information senders selectively communicate their own, biased, beliefs (Moussaïd et al., 2015). Therefore, although communication may transfer information about forgone payoffs of other alternatives, which could mitigate the hot stove effect (Denrell, 2007; Yechiam and Busemeyer, 2006), future research should explore the potential impact of active sharing of richer information on collective learning situations (Toyokawa et al., 2014).

In contrast, previous studies suggested that competitions or conflicts of interest among individuals can lead to better collective intelligence than fully cooperative situations (Conradt et al., 2013) and can promote adaptive risk taking (Arbilly et al., 2011). Further research will identify conditions under which cooperative communication containing richer information can improve decision making and drive adaptive cumulative cultural transmission (Csibra and Gergely, 2011; Morgan et al., 2015), when adverse biases in individual decision-making processes prevail.

The generality of our dynamics model should apply to various collective decision-making systems, not only to human groups. Because it is a fundamental property of adaptive reinforcement learning, risk aversion due to the hot stove effect should be widespread in animals (Real, 1981; Weber et al., 2004; Hertwig and Erev, 2009). Therefore, its solution, the collective behavioural rescue, should also operate broadly in collective animal decision making because frequency-based copying is one of the common social learning strategies (Hoppitt and Laland, 2013; Grüter and Leadbeater, 2014). Future research should determine to what extent the collective behavioural rescue actually impacts animal decision making in wider contexts, and whether it influences the evolution of social learning, information sharing, and the formation of group living.

We have identified a previously overlooked mechanism underlying the adaptive advantages of frequency-based social learning. Our results suggest that an informational benefit of group living could exist well beyond simple informational pooling where individuals can enjoy the wisdom of crowds effect (Ward and Zahavi, 1973). Furthermore, the flexibility emerging through the interaction of negative and positive feedback suggests that conformity could evolve in a wider range of environments than previously assumed (Aoki and Feldman, 2014; Nakahashi et al., 2012), including temporally variable environments (Aplin et al., 2017). Social learning can drive self-organisation, regulating the mitigation and amplification of behavioural biases and canalising the course of repeated decision making under risk and uncertainty.

## Materials and methods

### The baseline asocial learning model and the hot stove effect

We assumed that the decision maker updates their value of choosing the alternative i (∈{s,r}) at time t following the Rescorla–Wagner learning rule: $Q_{i,t+1}\leftarrow \left(1-\alpha \right)\,Q_{i,t}+\alpha \,\pi_{i,t}$, where α (0≤α≤1) is a *learning rate*, manipulating the step size of the belief updating, and $\pi_{i,t}$ is a realised payoff from the chosen alternative i at time t (Sutton and Barto, 2018). The larger the α, the more weight is given to recent experiences, making reinforcement learning more myopic. The Q value for the unchosen alternative is unchanged. Before the first choice, individuals had no previous preference for either option (i.e. $Q_{r,1}=Q_{s,1}=0$). Then Q values were translated into choice probabilities through a softmax (or multinomial-logistic) function such that $P_{i,t}=\exp\left(\beta \,Q_{i,t}\right)/\left(\exp\left(\beta \,Q_{s,t}\right)+\exp\left(\beta \,Q_{r,t}\right)\right)$, where β, the *inverse temperature*, is a parameter regulating how sensitive the choice probability is to the value of the estimate Q (i.e. controlling the proneness to explore).

In such a risk-heterogeneous multi-armed bandit setting, reinforcement learners are prone to exhibiting suboptimal risk aversion (March, 1996; Denrell, 2007; Hertwig and Erev, 2009), even though they could have achieved high performance in a risk-homogeneous task where all options have an equivalent payoff variance (Sutton and Barto, 2018). Denrell, 2007 mathematically derived the condition under which suboptimal risk aversion arises, depicted by the dashed curve in Figure 1b. In the main analysis, we focused on the case where the risky alternative had μ=1.5 and s.d.=1 and the safe alternative generated $\pi_{s}=1$ unless otherwise stated, that is, where choosing the risky alternative was the optimal strategy for a decision maker in the long run.

### Collective learning and social influences

We extended the baseline model to a collective learning situation in which a group of 10 individuals completed the task simultaneously and individuals could obtain social information. For social information, we assumed a simple frequency-based social cue specifying distributions of individual choices (McElreath et al., 2005; McElreath et al., 2008; Toyokawa et al., 2017; Toyokawa et al., 2019; Deffner et al., 2020). Following the previous modelling of social learning in such multi-agent multi-armed bandit situations (e.g. Aplin et al., 2017; Barrett et al., 2017; McElreath et al., 2005; McElreath et al., 2008; Toyokawa et al., 2017; Toyokawa et al., 2019; Deffner et al., 2020), we assumed that social influences on reinforcement learning would be expressed as a weighted average between the softmax probability based on the Q values and the conformist social influence, as follows:

(1) $$P_{i,t}=(1-\sigma )\frac{\exp(\beta Q_{i,t})}{\exp(\beta Q_{r,t})+\exp(\beta Q_{s,t})}+\sigma \frac{(N_{i,t-1}+0.1{)}^{\theta }}{(N_{s,t-1}+0.1{)}^{\theta }+(N_{r,t\%-1}+0.1{)}^{\theta }}$$

where σ was a weight given to the social influence (*copying weight*) and θ was the strength of conformist influence (*conformity exponent*), which determines the influence of social frequency on choosing the alternative i at time t-1, that is, $N_{i,t-1}$. The larger the conformity exponent θ, the higher the influence that was given to an alternative that was chosen by more individuals, with non-linear conformist social influence arising when θ>1. We added a small number, 0.1, to $N_{i,t-1}$ so that an option chosen by no one (i.e., $N_{i,t-1}=0$) could provide the highest social influence when θ<0 (negative frequency bias). Although this additional 0.1 slightly reduces the conformity influence when θ>0, we confirmed that the results were qualitatively unchanged. Note also that in the first trial t=1, we assumed that the choice was determined solely by the asocial softmax function because there was no social information available yet.

Note that when σ=0, there is no social influence, and the decision maker is considered an asocial learner. It is also worth noting that when σ=1 with θ>1, individual choices become fully contingent on the group’s most common behaviour, which was assumed in some previous models of strong conformist social influences in sampling behaviour (Denrell and Le Mens, 2017). The descriptions of the parameters are shown in Table 1. The simulations were run in R 4.0.2 (https://www.r-project.org) and the code is available at (the author’s github repository).

### The approximated dynamics model of collective behaviour

We assume a group of N individuals who exhibit two different behavioural states: choosing a safe alternative S, exhibited by $N_{S}$ individuals; and choosing a risky alternative R, exhibited by $N_{R}$ individuals ($N=N_{S}+N_{R}$). We also assume that there are two different ‘inner belief’ states, labelled ‘-’ and ‘+’. Individuals who possess the negative belief prefer the safe alternative S to R, while those who possess the positive belief prefer R to S. A per capita probability of choice shift from one behavioural alternative to the other is denoted by P. For example, $P_{S}^{-}$ means the individual probability of changing the choice to the safe alternative from the risky alternative under the negative belief. Because there exist $N_{S}^{-}$ individuals who chose S with belief -, the total number of individuals who ‘move on’ to S from R at one time step is denoted by $P_{S}^{-}N_{S}^{-}$. We assume that the probability of shifting to the more preferable option is larger than that of shifting to the less preferable option, that is, $P_{S}^{-}>P_{R}^{-}$ and $P_{R}^{+}>P_{S}^{+}$ (Figure 4a).

We assume that the belief state can change by choosing the risky alternative. We define that the per capita probability of becoming + state, that is, having a higher preference for the risky alternative, is e (0≤e≤1), and hence $N_{R}^{+}=e\,N_{R}$. The rest of the individuals who choose the risky alternative become - belief state, that is, $N_{R}^{-}=\left(1-e\right)\,N_{R}$.

We define ‘e’ so that it can be seen as a risk premium of the gambles. For example, imagine a two-armed bandit task equipped with one risky arm with Gaussian noises and the other a sure arm. The larger the mean expected reward of the risky option (i.e. the higher the risk premium), the more people who choose the risky arm are expected to obtain a larger reward than what the safe alternative would provide. By assuming e>1/2, therefore, it approximates a situation where risk seeking is optimal in the long run.

Here, we focus only on the population dynamics: If more people choose S, $N_{S}$ increases. On the other hand, if more people choose R, $N_{R}$ increases. As a consequence, the system may eventually reach an equilibrium state where both $N_{S}$ and $N_{R}$ no longer change. If we find that the equilibrium state of the population (denoted by *) satisfies $N_{R}^{⋆}>N_{S}^{⋆}$, we define that the population exhibits risk seeking, escaping from the hot stove effect. For the sake of simplicity, we assumed $p_{l}=P_{R}^{-}=P_{S}^{+}$ and $p_{h}=P_{R}^{+}=P_{S}^{-}$, where $0\leq p_{l}\leq p_{h}\leq 1$, for the asocial baseline model.

Considering $N_{R}^{+}=e\,N_{R}$ and $N_{R}^{-}=\left(1-e\right)\,N_{R}$, the dynamics are written as the following differential equations:

(2) $$\left\{ \begin{array}{ll} \frac{dN_{R}}{dt}=p_{l}N_{S}^{-}-p_{h}(1-e)N_{R}+p_{h}N_{S}^{+}-p_{l}eN_{R} & \\ \frac{dN_{S}^{-}}{dt}=-p_{l}N_{S}^{-}+p_{h}(1-e)N_{R}, & \\ \frac{dN_{S}^{+}}{dt}=-p_{h}N_{S}^{+}+p_{l}eN_{R}. & \end{array}\right.$$

Overall, our model crystallises the asymmetry emerging from adaptive sampling, which is considered as a fundamental mechanism of the hot stove effect (Denrell, 2007; March, 1996): Once decision makers underestimate the expected value of the risky alternative, they start avoiding it and do not have another chance to correct the error. In other words, although there would potentially be more individuals who obtain a preference for R by choosing the risky alternative (i.e. e>0.5), this asymmetry raised by the adaptive balance between exploration–exploitation may constantly increase the number of people who possess a preference for S due to underestimation of the value of the risky alternative. If our model is able to capture this asymmetric dynamics properly, the relationship between e (i.e. the potential goodness of the risky option) and $p_{l}/p_{h}$ (i.e. the exploration–exploitation) should account for the hot stove effect, as suggested by previous learning model analysis (Denrell, 2007). The equilibrium analysis was conducted in Mathematica (code is available online). The results are shown in Figure 4.

### Collective dynamics with social influences

For social influences, we assumed that the behavioural transition rates, $P_{S}$ and $P_{R}$, would depend on the number of individuals $N_{S}$ and $N_{R}$ as follows:

(3) $$\left\{ \begin{array}{ll} P_{S}^{-}=(1-\sigma )p_{h}+\sigma \frac{N_{S}^{\theta }}{N_{R}^{\theta }+N_{S}^{\theta }}, & \\ P_{R}^{-}=(1-\sigma )p_{l}+\sigma \frac{N_{R}^{\theta }}{N_{R}^{\theta }+N_{S}^{\theta }}, & \\ P_{S}^{+}=(1-\sigma )p_{l}+\sigma \frac{N_{S}^{\theta }}{N_{R}^{\theta }+N_{S}^{\theta }}, & \\ P_{R}^{+}=(1-\sigma )p_{h}+\sigma \frac{N_{R}^{\theta }}{N_{R}^{\theta }+N_{S}^{\theta }}, & \end{array}\right.$$

where σ is the weight of social influence and θ is the strength of the conformist bias, corresponding to the agent-based learning model (Table 1). Other assumptions were the same as in the baseline dynamics model. The baseline dynamics model was a special case of this social influence model with σ=0. Because the system was not analytically tractable, we obtained the numeric solution across different initial distribution of $N_{S,t=0}$ and $N_{R,t=0}$ for various combinations of the parameters.

### The online experiments

The experimental procedure was approved by the Ethics Committee at the University of Konstanz (‘Collective learning and decision-making study’). Six hundred nineteen English-speaking subjects [294 self-identified as women, 277 as men, 1 as other, and the rest of 47 unspecified; mean (minimum, maximum) age = 35.2 (18, 74) years] participated in the task through the online experimental recruiting platform Prolific Academic. We excluded subjects who disconnected from the online task before completing at least the first 35 rounds from our computational model-fitting analysis, resulting in 585 subjects (the detailed distribution of subjects for each condition is shown in Table 3). A parameter recovery test had suggested that the sample size was sufficient to reliably estimate individual parameters using a hierarchical Bayesian fitting method (see below; Figure 6—figure supplement 3).

#### Design of the experimental manipulations

The group size was manipulated by randomly assigning different capacities of a ‘waiting lobby’ where subjects had to wait until other subjects arrived. When the lobby capacity was 1, which happened at probability 0.1, the individual condition started upon the first subject’s arrival. Otherwise, the group condition started when there were more than three people at 3 min since the lobby opened (see Appendix 1 Supplementary Methods). If there were only two or fewer people in the lobby at this stage, the subjects each were assigned to the individual condition. Note that some groups in the group condition ended up with only two individuals due to a drop out of one individual during the task.

We used three different tasks: a 1-risky-1-safe task, a 1-risky-3-safe task, and a 2-risky-2-safe task, where one risky option was expected to give a higher payoff than other options on average (that is, tasks with a positive risk premium [positive RP]). To confirm our prediction that risky shift would not strongly emerge when risk premium was negative (i.e. risk seeking was suboptimal), we also conducted another 1-risky-1-safe task with a negative risk premium (the negative RP task). Participants’ goal was to gather as many individual payoff as possible, as monetary incentives were given to the individual performance. In the negative RP task, risk aversion was favourable instead. All tasks had 70 decision-making trials. The task proceeded on a trial basis; that is, trials of all individuals in a group were synchronised. Subjects in the group condition could see social frequency information, namely, how many people chose each alternative in the preceding trial. No social information was available in the first trial. These tasks were assigned randomly as a between subject condition, and subjects were allowed to participate in one session only.

We employed a skewed payoff probability distribution rather than a normal distribution for the risky alternative, and we conducted not only a two-armed task but also four-armed bandit tasks, because our pilot study had suggested that subjects tended to have a small susceptibility to the effect ($\alpha_{i}\,\left(\beta_{i}+1\right)\ll 2$), and hence we needed more difficult settings than the conventional Gaussian noise binary-choice task to elicit risk aversion from individual decision makers. Running agent-based simulations, we confirmed that these task setups used in the experiment could elicit the collective rescue effect (Figure 1—figure supplement 5 Figure 1—figure supplement 6).

The details of the task setups are as follows:

##### The 1-risky-1-safe task (positive RP)

The optimal risky option produced either 50 or 550 points at probability 0.7 and 0.3, respectively (the expected payoff was 200). The safe option produced 150 points (with a small amount of Gaussian noise with s.d. = 5).

##### The 1-risky-3-safe task (positive RP)

The optimal risky option produced either 50 or 425 points at probability 0.6 and 0.4, respectively (the expected payoff was 200). The three safe options each produced 150, 125, and 100 points, respectively, with a small Gaussian noise with s.d. = 5.

##### The 2-risky-2-safe task (positive RP)

The optimal risky option produced either 50 or 425 points at probability 0.6 and 0.4, respectively (the expected payoff was 200). The two safe options each produced 150 and 125 points, respectively, with a small Gaussian noise with s.d. = 5. The suboptimal risky option, whose expected value was 125, produced either 50 or 238 points at probability 0.6 and 0.4, respectively.

##### The 1-risky-1-safe task (negative RP)

The setting was the same as in the 1-risky-1-safe positive RP task, except that the expected payoff from the risky option was smaller than the safe option, producing either 50 or 220 points at probability 0.7 and 0.3, respectively (the expected payoff was 101).

We have confirmed through agent-based model simulations that the collective behavioural rescue could emerge in tasks equipped with the experimental settings (Figure 1—figure supplement 5). We have also confirmed that risk seeking does not always increase when risk premium is negative (Figure 1—figure supplement 6). With the four-armed tasks we aimed to demonstrate that the rescue effect is not limited to binary-choice situations. Other procedures of the collective learning task were the same as those used in our agent-based simulation shown in the main text. The experimental materials including illustrated instructions can be found in Video 1 (individual condition) and Video 2 (group condition).

**Video 1.** A sample screenshot of the online experimental task (Individual condition). This video was taken only for the demonstration purpose and hence not associated to any actual participant’s behaviour.

**Video 2.** A sample screenshot of the online experimental task with N = 3 (group condition). This video was taken only for the demonstration purpose and hence not associated to any actual participant’s behaviour. Also note that actual participants could see only one browser window per participant in the experimental sessions.

### The hierarchical Bayesian model fitting

To fit the mixed logit model (GLMM) as well as the learning model, we used a hierarchical Bayesian method. For the learning model, we estimated the global means ($\mu_{\alpha }$, $\mu_{\beta }$, $\mu_{\sigma }$, and $\mu_{\theta }$) and global variances ($v_{\alpha }$, $v_{\beta }$, $v_{\sigma }$, and $v_{\theta }$) for each of the four experimental conditions and for the individual and group conditions separately. For the individual condition, we assumed σ=0 for all subjects and hence no social learning parameters were estimated. Full details of the model-fitting procedure and prior assumptions are shown in the Supplementary Methods. The R and Stan code used in the model fitting are available from an online repository.

#### The GLMM

We conducted a mixed logit model analysis to investigate the relationship between the proportion of choosing the risky option in the second half of the trials ($P_{r,t>35}$) and the fit learning parameters ($\alpha_{i}\,\left(\beta_{i}+1\right)$, $\sigma_{i}$, and $\theta_{i}$). Since no social learning parameters exist in the individual condition, the dummy variable of the group condition was considered ($G_{i}=1$ if individual i was in the group condition or 0 otherwise). The formula used is $logit(P_{r,t>35})$ = $\gamma_{0}+\gamma_{1}\alpha_{i}(\beta_{i}+1)+\gamma_{2}G_{i}+\gamma_{3}G_{i}\alpha_{i}(\beta_{i}+1)+\gamma_{4}G_{i}\sigma_{i}+\gamma_{5}G_{i}\theta_{i}+\epsilon_{i}+\epsilon_{g}$, where $\epsilon_{i}$ and $\epsilon_{g}$ were the random effect of individual and group, respectively. The model fitting using the Markov chain Monte Carlo (MCMC) method was the same as what was used for the computational model fitting, and the code are available from the repository shown above.

#### Model and parameter recovery, and post hoc simulation

To assess the adequacy of the hierarchical Bayesian model-fitting method, we tested how well the hierarchical Bayesian method (HBM) could recover ‘true’ parameter values that were used to simulate synthetic data. We simulated artificial agents’ behaviour assuming that they behave according to the social learning model with each parameter setting. We generated ‘true’ parameter values for each simulated agent based on both experimentally fit global parameters (Table 1; parameter recovery test 1). In addition, we ran another recovery test using arbitrary global parameters that deviated from the experimentally fit values (parameter recovery test 2), to confirm that our fitting procedure was not just ‘attracted’ to the fit value. We then simulated synthetic behavioural data and recovered their parameter values using the HBM described above. Both parameter recovery tests showed that all the recovered individual parameters were positively correlated with the true values, whose correlation coefficients were all larger than 0.5. We also confirmed that 30 of 32 global parameters in total were recovered within the 95% Bayesian credible intervals, and that even those two non-recovered parameters ($\mu_{\beta }$ for the 2-risky-2-safe task in parameter recovery test 1 and $\mu_{\alpha }$ for the 1-risky-3-safe task in parameter recovery test 2) did not deviate so much from the true value (Figure 6—figure supplement 3).

We compared the baseline reinforcement learning model, the decision-biasing model, and the value-shaping model (see Supplementary Methods) using Bayesian model selection (Stephan et al., 2009). The model frequency and exceedance probability were calculated based on the Widely Applicable Information Criterion (WAIC) values for each subject (Watanabe and Opper, 2010). We confirmed accurate model recovery by simulations using our task setting (Figure 6—figure supplement 2).

We also ran a series of individual-based model simulations using the calibrated global parameter values for each condition. First, we randomly sampled a set of agents whose individual parameter values were drawn from the fit global parameters. Second, we let this synthetic group of agents perform the task for 70 rounds. We repeated these steps 100,000 times for each task setting and for each individual and group condition.

## Data Availability

Code for the agent-based simulation as well as for the experimental data analyses can be found in the main author's Github repository https://github.com/WataruToyokawa/ToyokawaGaissmaier2021 (copy archived at swh:1:rev:6fca0b26c33af3a5b3c415719fa3df0dced15149).

## Additional files

Transparent reporting form

## Appendix 1

### Supplementary methods

#### An analytical result derived by Denrell, 2007

In the simplest setup of the two-armed bandit task, Denrell, 2007 derived an explicit form for the asymptotic probability of choosing the risky alternative $P_{r}^{⋆}$ (as t→∞) as follows:

(4) $$P_{r}^{⋆}=\frac{1}{1+\exp\left[\frac{\alpha \beta^{2}{\text{s.d.}}^{2}}{2(2-\alpha )}-\beta (\mu -\pi_{s})\right]}.$$

Equation 4 identifies a condition under which reinforcement learners exhibit risk aversion. In fact, when there is no risk premium (i.e. $\mu \leq \pi_{s}$), the condition of risk aversion always holds, that is, $P_{r}^{⋆}<0.5$. Consider the case where risk aversion is suboptimal, that is, $\mu >\pi_{s}$. Equation 4 suggests that suboptimal risk aversion emerges when learning is myopic (i.e. when α is large) and/or decision making is less explorative (i.e. when β is large). For instance, when the payoff distribution of the risky alternative is set to $\mu =\pi_{s}+0.5$ and ${\text{s.d.}}^{2}=1$, the condition of risk aversion, $P_{r}^{⋆}<0.5$, holds under β>(2−α)/α, which corresponds to the area above the dashed curve in Figure 1b in the main text. Risk aversion becomes more prominent when the risk premium $\mu -\pi_{s}$ is small and/or the payoff variance ${\text{s.d.}}^{2}$ is large.

#### The online experiments

##### Subjects

The positive risk premium (positive RP) tasks were conducted between August and October 2020 (recruiting 492 subjects), while the negative risk premium (negative RP) task was conducted in September 2021 (recruiring 127 subjects) in response to the comments from peer reviewers. All subjects declared their residence in the United Kingdom, the United States, Ireland, or Australia. All subjects consented to participation through an online consent form at the beginning of the task. We excluded subjects who disconnected from the online task before completing at least the first 35 rounds from our computational model-fitting analysis, resulting in 467 subjects for the positive RP tasks and 118 subjects for the negative RP task (the detailed distribution of subjects for each condition is shown in Table 1 in the main text). The task was available only for English-speaking subjects and they had to be 18 years old or older. Only subjects who passed a comprehension quiz at the end of the instructions could enter the task. Subjects were paid 0.8 GBP as a show-up fee as well as an additional bonus payment depending on their performance in the decision-making task In the positive RP tasks 500 artificial points were converted to 8 pence, while in the negative RP task 500 points were converted to 10 pence so as to compensate the less productive environment, resulting in a bonus ranging between £1.0 and £3.5.

##### Sample size

Our original target sample size for the positive RP tasks was 50 subjects for the individual condition and 150 subjects for the group condition where our target average group size was 5 individuals per group. For the negative RP task, we aimed to recruit 30 individuals for the individual condition and 100 individuals (that is, 20 groups of 5) for the group condition. Subjects each completed 70 trials of the task. The sample size and the trial number had been justified by a model recovery analysis of a previous study (Toyokawa et al., 2019).

Because of the nature of the ‘waiting lobby’, which was available only for 3 min, we could not fully control the exact size of each experimental group. Therefore, we set the maximum capacity of a lobby to 8 individuals for the 1-safe-1-risky task, which was conducted in August 2020, so as to buffer potential dropouts during the waiting period. Since we learnt that dropping out happened far less than we originally expected, we reduced the lobby capacity to 6 for both the 1-risky-3-safe and the 2-risky-2-safe task, which were conducted in October 2020. As a result, we had 20 groups (mean group size = 6.95), 21 groups (mean group size = 4.7), 19 groups (mean group size = 4.3), and 21 gorups (mean group size = 4.4), for the 1-risky-1-safe, 1-risky-3-safe, 2-risky-2-safe task, and the negative risk premium 2-armed task, respectively. Although we could not achieve the sample size targeted, partly due to the dropouts during the task and to a fatal error occurring in the experimental server in the first few sessions of the four-armed tasks, the parameter recovery test with N=105 suggested that the current sample size should be reliable enough to estimate social influences for each subject (Figure 6—figure supplement 3).

#### The hierarchical Bayesian parameter estimation

We used the hierarchical Bayesian method (HBM) to estimate the free parameters of our learning model. HBM allowed us to estimate individual differences, while this individual variation is bounded by the group-level (i.e. hyper) parameters. To do so, we used the following non-centred reparameterisation (the ‘Matt trick’) as follows:

$$\text{logit}(\alpha_{i})=\mu_{\alpha }+v_{\alpha }*\alpha_{\text{raw},i}$$

where $\mu_{\alpha }$ is a global mean of $\text{logit}\,\left(\alpha_{i}\right)$ and $v_{\alpha }$ is a global scale parameter of the individual variations, which is multiplied by a standardised individual random variable $\alpha_{\text{raw},i}$. We used a standardised normal prior distribution centred on 0 for $\mu_{\alpha }$ and an exponential prior for $v_{\alpha }$. The same method was applied to the other learning parameters $\beta_{i}$, $\sigma_{i}$, and $\theta_{i}$.

We assumed that the ‘raw’ values of individual random variables ($\alpha_{\text{raw},i}$, $\beta_{\text{raw},i}$, $\sigma_{\text{raw},i}$, $\theta_{\text{raw},i}$) were drawn from a multivariate normal distribution. The correlation matrix was estimated using a Cholesky decomposition with a weakly informative Lewandowski–Kurowicka–Joe prior that gave a low likelihood to very high or very low correlations between the parameters (McElreath, 2020; Deffner et al., 2020).

##### Model fitting

All models were fitted using the Hamiltonian Monte Carlo engine CmdStan 2.25.0 (https://mc-stan.org/cmdstanr/index.html) in R 4.0.2 (https://www.r-project.org). The models contained at least six parallel chains and we confirmed convergence of the MCMC using both the Gelman–Rubin statistics criterion $\hat{R}\leq 1.01$ and the effective sample sizes greater than 500. The R and Stan code used in the model fitting are available from an online repository.

#### The value-shaping social influence model

We considered another implementation of social influences in reinforcement learning, namely, a value-shaping (Najar et al., 2020) (or ‘outcome-bonus’ Biele et al., 2011) model rather than the decision-biasing process assumed in our main analyses. In the value-shaping model, social influence modifies the Q value’s updating process as follows:

$$Q_{i,t+1}\leftarrow (1-\alpha )Q_{i,t}+\alpha \left(\pi_{i,t}+\sigma_{vs}\bar{\pi }\frac{N_{i,t-1}^{\theta }}{N_{s,t-1}^{\theta }+N_{r,t-1}^{\theta }}\right)$$

where the social frequency cue acts as an additional ‘bonus’ to the value that was weighted by $\sigma_{v\,s}$ ($\sigma_{vs}>0$) and standardised by the expected payoff from choosing randomly among all alternatives $\bar{\pi }$. Here we assumed no direct social influence on the action selection process (i.e., σ=0 in our main model). We confirmed that the collective behavioural rescue could emerge when the inverse temperature β was sufficiently small (Figure 1—figure supplement 2). Although it is beyond the focus of this article whether any other types of models would fit better with human data than the models we considered in this study, it is an interesting question for future research. For such an attempt, see Najar et al., 2020.
