# Supplementary material for: Conformist social learning leads to self-organised prevention against adverse bias in risky decision making
Source: eLife. 2022 May 10;11:e75308. doi: 10.7554/eLife.75308 (PMC9090329; doi:10.7554/eLife.75308)
Supplement: Peer review history

# Editor report: Editor's evaluation

**DOI:** 10.7554/eLife.75308.sa0

**Authors:** Mimi Liljeholm

**Affiliations:** https://ror.org/04gyf1771 University of California, Irvine United States

The authors use reinforcement learning and dynamic modeling to formalize the favorable effects of conformity on risk taking, demonstrating that social influence can produce an adaptive risk-seeking equilibrium at the population level. The work provides a rigorous analysis of a paradoxical interplay between social and economic choice.

# Decision letter

**DOI:** 10.7554/eLife.75308.sa1

**Affiliations:** https://ror.org/04gyf1771 University of California, Irvine United States

In the interests of transparency, eLife publishes the most substantive revision requests and the accompanying author responses.

**Decision letter after peer review:**

[Editors’ note: the authors submitted for reconsideration following the decision after peer review. What follows is the decision letter after the first round of review.]

Thank you for submitting the paper "Conformist social learning leads to self-organised prevention against adverse bias in risky decision making" for consideration by *eLife*. Your article has been reviewed by 3 peer reviewers, including Mimi Liljeholm as the Reviewing Editor and Reviewer #3, and the evaluation has been overseen by a Senior Editor.

We are sorry to say that, after consultation with the reviewers, we have decided that this work will not be considered further for publication by *eLife*.

There was consensus among the reviewers that the paper addresses an important and impactful topic. The influence of conformity on economic choice is still largely unexplored, and the rigorous modeling approach employed here is valuable. However, a primary weakness is the lack of integration with the relevant empirical and theoretical context, which imperils both the novelty and interpretability of the results:

First, it is unclear whether these findings constitute enough of an advance over those reported by Denrell and Le Mens (2007) to warrant publication in *eLife*. Second, there is no effort to incorporate processes supporting normative and informational conformity into the models. Notably, these issues are somewhat connected, in that a formal integration of normative and informational conformity with sampling-based collective rescue might go a long way towards distinguishing this work from that of Denrell and Le Mens. Thus, while these issues are too open-ended for a revision decision at *eLife*, the enthusiasm among reviewers was such that, should these concerns be fully addressed, the paper might be considered again as a new submission.

The specific comments from the reviewers are appended below for further reference.

*Reviewer #1:*

In this study the authors investigated the effect of social influence on individuals' risk aversion in a 2-armed bandit task. Using a reinforcement learning model and a dynamic model they showed that social influence can in fact diminish risk aversion. The authors then conducted a series of online experiments and found that their experimental findings were consistent with the prediction of their simulations.

The authors addressed an important question in the literature and adopted an interesting approach by first making predictions using simulations and then verifying those predictions with experimental data. The modelling has been conducted very carefully.

However, I have some concerns about the interpretation of the findings which might be addressed using additional analysis and or rewriting some parts of the manuscript. The study does not clarify whether in this task participants copy others to maximize their accuracy (informational conformity) or alternatively to be aligned with others (normative conformity). It is possible that participants became riskier because most of the group were choosing the riskier decisions (regardless of the outcome). In addition to that, an earlier study showed that people make riskier decisions when they make decision alongside other people. This might be a potential confound of this study.

One potentially interesting design would be to test people in a situation where only the minority of the group members choose the optimal option (riskier option). If participants' choices become riskier even in this condition, we can conclude that they were not just copying the majority, but were maximising their reward by observing others' decisions and outcomes.

In this study the authors investigated the effect of social influence on individuals' risk aversion in a 2-armed bandit task. Using a reinforcement learning model and a dynamic model they showed that social influence can in fact diminish risk aversion. The authors then conducted a series of online experiments and observed the same effect in their experimental data as well. The research question is timely, and the modelling has been done carefully. However, I have some comments and concerns about the interpretations of their findings.

– My first question is do the participants copy others because the other risky option sounds better in terms of reward or because they copy others just because being in alignment with others is rewarding? This brings us to the distinction between informational and normative influence. For example, a recent study showed that copying others is not necessarily motivated by maximising accuracy (Mahmoodi et al. 2018, see also Cialdinin and Goldstein 2004). In their experimental data, the authors found that participants do not copy others (choosing the risky options) as much as they should do. Does it suggest that their conformity toward others cannot be fully explained by informational motives (where the aim of conformity is to maximise payoff/accuracy). I suggest that the authors discuss each of these possibilities and then explain to which of these two types of influence their findings belong to.

– An earlier study showed that people's decisions become riskier when they make decisions with others (Bault et al. PNAS 2011). Could this explain the findings that are presented in this paper? Can the models distinguish between these two types of change in behaviour? I strongly suggest the authors to discuss the Bault et al. paper and discuss how their findings deviate from this study.

– In one section the authors show that reducing heterogeneity in groups undermines group performance. It brought my attention to a study (Lorenz et al. PNAS 2011) which suggested that social influence can undermine wisdom of crowds through reducing heterogeneity of opinions. It seems that the authors are presenting the same phenomenon as that suggested by Lorenz and colleagues. I suggest that the authors cite that study and discuss how their results is related to that study and whether their findings broaden our understanding of the effect of heterogeneity on collective performance.

– Nothing can be found about the model in the main text. Similarly, some of the terms are not even defined before they are used in the main text. For example, the term "asocial learning" is only defined in the Figure 2 caption. I suggest that the authors briefly explain the model and the key terms in the main text before presenting the result. I also strongly suggest that the authors mention in the main text that the detail of the model is presented in the methods.

In the introduction reads: social influence does not mindlessly increase risk seeking; instead, it may work only when to do so is adaptive. I believe this sentence is vague in its current form. I suggest that the authors elaborate on it, especially on the last part (i.e. to do so is adaptive).

*Reviewer #2:*

The paper considers an interesting puzzle. While most psychological studies of conformity tend to focus on negative effects, animal research highlights positive effects of conformity. The current analysis tries to clarify this apparent puzzle by clarifying one positive effect of conformity: Reduction of the hot stove effect that impairs maximization when taking risk is optimal.

The paper can be improved by building on previous efforts (e.g., Denrell and Le Mens, 2007) to clarify the impact of social influence on the hot stove effect. It would also be good to try to simplify the model, and use the same tasks in the theoretical analysis and the experimental study.

One interesting open question involves the impact of the increase in the information, available today (in social networks like Facebook) concerning the behavior of other individuals. I think that the current analysis predicts an increase in risk taking.

The paper considers an interesting puzzle. While most psychological studies of conformity tend to focus on negative effects, animal research highlights positive effects of conformity. The current analysis tries to clarify this apparent puzzle by clarifying one positive effect of conformity: Reduction of the hot stove effect that impairs maximization when taking risk is optimal.

The paper's main shortcoming is the fact that it is difficult to understand how it adds to the observations presented in Denrell and Le Mens (2007), and more recent research by Le Mens. It is possible that the authors can address this shortcoming by clarifying the difference between pure conformity (or imitation) and the impact of social influence examined by Le Mens and his co-authors.

Another shortcoming involves the difference between the choice task analyzed in the theoretical analysis, and the task examined in the experiment. The theoretical analysis focuses on normal distributions, and the experiment focuses on asymmetric bimodal distributions. The authors suggest that they chose to switch to asymmetric bimodal distributions as the hot stove effect, exhibited by human subjects, in the case of normal distributions is not strong. If this is the case, it would be good to adjust the theoretical model and use a model that better capture human behavior.

A third shortcoming involves the complexity to the theoretical model. Since this model is only used to demonstrate that conformity can reduce the hot stove effect, and is not supposed to capture the exact magnitude of the two effects, I could not understand why it includes so many parameters. For example, it would be nice to add only one parameter to the basic reinforcement learning model. If more parameters are needed it would be good to show why they are needed.

*Reviewer #3:*

The authors use reinforcement learning and dynamic modeling to formalize the favorable effects of conformity on risk taking, demonstrating that social influence can produce an adaptive risk-seeking equilibrium at the population level. The work provides a rigorous analysis of a paradoxical interplay between social and economic choice.

Conformity is commonly attributed to either an intrinsic reward of group membership, or to inferences about the optimality of others' behavior (i.e., normative vs. informational). Neither of these aspects of conformity are addressed here, which limits the interpretability of the results. For example, if there is an intrinsic reward associated with majority alignment, that should contribute to the reinforcement of such decisions; moreover, inferences about the optimality of observed behavior likely change from early trials, in which others can be assumed to simply explore, to later trials, in which the decisions of others may be indicative of their success. The work would be more impactful if it considered how these factors might affect the potential for collective rescue.

An interesting question is whether a substantial payoff contingent on choosing a risky option may server to reinforce the act of risk taking itself, and how such processes might propagate social influence across environments.

I suspect that the paper was initially written with the Methods following the Introduction, and with the Methods being subsequently moved to the end without much additional editing. As a result, very few of the variables and concepts (e.g., conformist influence, copying weight, positive/negative feedback) are defined in the main text upon first mention, which makes for extremely onerous reading.

Cases where the risky option yields a lesser mean payoff, producing a potentially detrimental social influence, should be given full weight in the main text, and should have been included in the behavioral study. Generally, the discrepancy between modeling and behavioral results is a bit disappointing. It is unclear why the behavioral experiment was not designed so as create the most relevant conditions.

My greatest concern is that the work does not integrate properly with its theoretical and empirical context. Additional analyses assessing the relative contributions of normative and informational conformity to socially induced risk-seeking would be helpful.

[Editors’ note: further revisions were suggested prior to acceptance, as described below.]

Thank you for resubmitting your work entitled "Conformist social learning leads to self-organised prevention against adverse bias in risky decision making" for further consideration by *eLife*. Your revised article has been evaluated by Michael Frank (Senior Editor) and a Reviewing Editor.

The manuscript has been improved, and Reviewer 2 recommends acceptance at this point, but Reviewer 3 has some remaining concerns, summarized below. We invite you to address all remaining concerns in a second round of revisions. Make sure to include point-by-point replies to each of Reviewer 3's recommendations.

1) Although the organization and writing is improved, it has some ways to go before the manuscript is ready for publication. For example, the basic aims and methods should be stated in one or two sentences at the end of the first, or at most second, paragraph of the introduction, giving the reader a clear sense of where things are going. Moreover, the "Agent-based model" section should be shortened (to include only what is needed to conceptually understand the model, leaving details for a table and the methods) and better integrated with the introduction, rather than inserted as (what appears to be) a super-section.

2) Just as the intro should include a concise description of the model, it should highlight the online experiments, and how they relate to the modeling.

3) The result section should start with a paragraph outlining the various hypotheses and corresponding analyses (i.e., a "roadmap" for the section).

4) Please quantify the performance of your model relative to others with formal comparisons (e.g., Bayesian Model Selection).

5) Please quantify all claims of associations with effect sizes and clearly justify all parameter cut-offs/values.

6) Streamline figures and included predicted/observed result plots wherever possible.

*Reviewer #3:*

The authors showed that when individuals learn about how they should decide in a situation where they can choose between a risky and a safe option, they might overcome maladaptive biases (e.g. exaggerated risk-aversion when risk-taking would be beneficial) by conforming with group behaviour. Strengths include rigorous and innovative computational modeling, a weakness might be that the set-up of the empirical study did not actually widely provoke behavioral phenomena at question, e.g. social learning (which is arguably at the core of the research question). Even though I am reviewing a revised manuscript I would hope the authors find a way to further improve clarity in the presentation of their research question and results.

My main concern was that even in the revised version I read, I found the paper not as accessible as I think it should be for a wide readership of a journal like *eLife*; and often times I found that things you could communicate in a straightforward way are put too complicated/expressed very verbosely. When reading the previous reviews after having read the revised paper, I also got the feeling that there were some misunderstandings. You clarified these specific points well in your responses, I think, but the lack in clarity might be even more drastic with an interdisciplinary readership this journal aims at as compared to the experts the journal has recruited now for this review? I will try to give some examples below.

The introduction should, imho provide a general intro to the question of the paper and how you've arrived to ask that question, avoiding too much technical jargon. After having read the paper, I realized that the research question is pretty straight-forward (and interesting) and derived from 1/2 previous observations, but this didn't become clear on the very first read.

Just as an example, some of the first sentences are…

"One rationale behind this optimistic view might come from the assumption that individuals tend to prefer a behavioural option that provides larger net benefits in utility over those providing lower outcomes. Therefore, even though uncertainty makes individual decision-making fallible, statistical filtering through informational pooling may be able to reduce uncertainty by cancelling out such noise."

This is only 1 example (it is something I noted throughout the paper… and also came up in the previous round of reviews) where I think these 2 sentences require some if not a little more background in decision-making (net benefits, utility, uncertainty, noise, stat filtering, informational pooling) to be understandable.

Other terminology like e.g. collective illusion, opportunity costs, description-based vs. experienced-based risk-taking paradigms, frequency-based influences, would be nice to be either defined in the text or replaced by a more accessible description in the introduction.

I know it is sometimes hard to mentalize which terminology others not working on the same things might struggle with, but given that this is an interdisciplinary journal, might it perhaps make sense to ask a researcher friend who is not exactly working on this topic to give it a read?

"such a risk-taking bias constrained by the fundamental nature of learning may function independently from the adaptive risk perception (Frey et al., 2017), potentially preventing adaptive risk taking."

Unclear without knowing or looking up the Frey paper – shorten ("to be too risk-averse might be maladaptive in some contexts"?) or explain.

Line 74-82 extremely long sentence – I think can easily be simplified? – "previous studies have neglected contexts where individuals learn about the environment both by own and others' experiences"

Line 84-89 is really long, too.

I would have been interested to learn more about the online experiments in the intro.

I do understand the reasoning of copy and pasting the agent Based Model description after having read the previous reviews, but it confused me when reading the article first; I think it needs to be integrated with Intro/Results section better (the first paragraph reads like intro/background still, but then it is about the authors' current study). I fear that readers don't know where they are in the paper at that point. (I much prefer journals with the Methods-Results order rather than the one *eLife* uses, as this would naturally circumvent this problem, but that's the challenge here, I reckon.)

Results

Subheadings: Make clearer which is the section that describes simulations and which is the empirical section.

Can you maybe start with reiterating in a structured way which parameters you set to which values and why in the simulation before describing the effects of it, how many trials you simulate (you start speaking of elongated time horizons but do not mention the original horizon length other than in the figure legend?) etc?

I know the Najar work and I think it is cool that you can generalize your results also to a value-based framework, but I do not think readers that do not know the Najar study will be able to follow this at it is described now, which makes it more confusing than interesting. So either elaborate what this means (accessible to non-initiated readers) or ban to the Supplement (would be a shame).

Would the value-based model fit the empirical data better?

When reading the first part of the Results section I constantly wondered: How did the group behave / How was the behaviour of the group determined in the simulation? Was variability considered? (this is something that's been manipulated in some empirical studies building on descriptive risk scenarios, e.g. Suzuki et al). It becomes clear when reading on/looking at Figure 3, but it is such a crucial point that it needs to be made clear from the beginning.

I think it is a major limitation that in the empirical study actual social learning was extremely limited, given that the paper claims to provide a formal account of the function of social learning in this situation?…. I would have thought that indeed trying to provoke more use of social influence by altering the experimental setup in a way the authors propose in their discussion would have been important, and given that this can be done online, also a feasible option.

Also, empirically, susceptibility to the hot stove effect, i.e., *α_i_*(*β_i_* + 1), seems to be very low – zero for most of the participants in some scenarios according to Figure 6A,B,C? – isn't this concerning, given that this is at the core of what the authors want to explain?

"those with a higher value of the susceptibility to the hot stove effect (*αi*(*βi* + 1)) were less likely to choose the risky alternative, whereas those who had a smaller value of *α_i_*(*β_i_* + 1) had a higher chance of choosing the safe alternative (Figure 6a-c), " -- I'm confused- should it read "those who had a smaller value of *α_i_*(*β_i_* + 1) had a higher chance of choosing the *risky* alternative"?

Could you please quantify this correlation in terms of an effect size? The association is not linear, from the Figure? Please specify.

Is the association driven by α or by β or really by the product?

"The behaviour in the group condition supports our theoretical predictions. In the PRP tasks, the proportion of choosing the favourable risky option increased with social influence (*σ_i_*) particularly for individuals who had a high susceptibility to the hot stove effect. On the other hand, social influence had little benefit for those who had a low susceptibility to the hot stove effect (e.g., *α_i_*(*β_i_* + 1) {less than or equal to} 0.5)." Can you quantify this with statistically (effect sizes etc)?

Did you do any form of model selection on the empirical data (with different set ups of your models, the reduced models (e.g. without σ), or the Najar type of model) to demonstrate that it is really your theoretically proposed model that fits the data best (e.g. Bayesian Model Selection)? Please include in the main manuscript. See Palminteri, TiCS on why this might be important.

I think Figure 6 is really overloaded. The legend somehow looks as it would belong only to panel c? The coloured plots are individual data points as a function of group size (which is what?) or copying weight? For me, in the current formatting, dots were too small to detect a continuous colour coding scheme (only yellow vs purple). The solid lines are simulated data? Can you show a regression line for the empirical data to allow for comparisons? Does it not differ substantially from the model predictions? I suggest making different plots for different purposes (compare predicted behaviour to empirical behaviour, show effect of copying weight, show effect of group size etc, show simulation were you plug in $\bar{\sigma }$>0.4)

"In keeping with this, if we extrapolated the larger value of the copying of weight (i.e., $\bar{\sigma_{i}}$> 0.4) into the best fitting social learning model with the other parameters calibrated, a strong collective rescue became prominent " – sorry, where does the value $\bar{\sigma }$>0.4 exactly come from for this analysis? Please give more detail/contextualise better.

Please try to be consistent with terminology *αi*(*βi* + 1) is sometimes called 'susceptibility ' or 'susceptibility value', which might be confusing, given that in some published articles susceptibility refers to susceptibility to social influence which would be another parameter…. I suggest to go through the manuscript once more and strictly only use one term for each parameter (the one you introduce in the table).

# Reply: Author response

**DOI:** 10.7554/eLife.75308.sa2

[Editors’ note: the authors resubmitted a revised version of the paper for consideration. What follows is the authors’ response to the first round of review.]

The specific comments from the reviewers are appended below for further reference.

Reviewer #1:

In this study the authors investigated the effect of social influence on individuals' risk aversion in a 2-armed bandit task. Using a reinforcement learning model and a dynamic model they showed that social influence can in fact diminish risk aversion. The authors then conducted a series of online experiments and found that their experimental findings were consistent with the prediction of their simulations.

The authors addressed an important question in the literature and adopted an interesting approach by first making predictions using simulations and then verifying those predictions with experimental data. The modelling has been conducted very carefully.

However, I have some concerns about the interpretation of the findings which might be addressed using additional analysis and or rewriting some parts of the manuscript.

The study does not clarify whether in this task participants copy others to maximize their accuracy (informational conformity) or alternatively to be aligned with others (normative conformity). It is possible that participants became riskier because most of the group were choosing the riskier decisions (regardless of the outcome).

We agree to the reviewer’s point that both informational and normative motivations would underlie conformity behaviour. It is correct that our model does not specify underlying individual motivations. In other words, the model does not depend on whether there are normative motivations to align with the majority or there exist only informational motivations for conformity. The result of our model can hold irrespective of them. Whatever the proximate reasons behind the conformist social influence, as long as individual choices are influenced by many others’ behaviour, experiences from the (otherwise avoided) risky alternative can increase, which results in mitigation of the hot stove effect. To make this point clearer, we have added texts in the Model section in the main text as follows:

– (Line 159 – 167): “A payoff realised was independent of others’ decisions and it was drawn solely from the payoff probability distribution specific to each alternative, thereby we assume neither direct social competition over the monetary reward (Giraldeau & Caraco, 2000) nor normative pressures towards majority alignment (Cialdini & Goldstein, 2004; Mahmoodi et al., 2018). The value of social information was assumed to be only informational (Nakahashi, 2012). Nevertheless, our model could apply to the context of normative social influences, because what we assumed here was modifications in individual choice probabilities due to social influences, irrespective of underlying motivations of conformity.”

For the sake of simplicity, in the online experiment we aimed to limit the underlying motivation of using social information to the informational one. Therefore, participants in our experiment had no direct monetary incentives for aligning with the majority and were kept anonymous during the task, which we thought minimised the possibility to evoke the normative motivation. Nevertheless, we agree with the reviewer’s point that the distinction between informational and normative conformity is important in a broader context of social influence, and both types of motivations could have in fact co-worked in the experiment as well as in many real-world situations. We added discussions to elaborate this point (see below). In particular, we expect that the weight for social learning (that is,σparameter in our model) would increase if both informational and normative motivations for conformity operate together, which would either promote more robust rescue effect ifσis still not too large, or trigger maladaptive herding (i.e. collective illusion) ifσbecomes too large. The discussion we have added are as follows:

– (Lines 572 – 586): “The weak reliance on social learning, which affected only about 15% of decisions, was unable to facilitate strong positive feedback. The little use of social information might have been due to the lack of normative motivations for conformity and to the stationarity of the task. In a stable environment, learners could eventually gather enough information as trials proceeded, which might have made them less curious about information gathering including social learning (Rendell et al., 2010). In reality, people might use more sophisticated social learning strategies whereby they change the reliance on social information flexibly over trials (Deffner et al., 2020; Toyokawa et al., 2017, 2019). Future research should consider more strategic use of social information, and will look at the conditions that elicit heavier reliance on the conformist social learning in humans, such as normative pressures for aligning with majority, volatility in the environment, time pressure, or an increasing number of behavioural options (Muthukrishna et al., 2015), coupled with larger group sizes (Toyokawa et al., 2019).”

In addition to that, an earlier study showed that people make riskier decisions when they make decision alongside other people. This might be a potential confound of this study.

We now cite some earlier studies, highlighting how our approach and the previous literature differ qualitatively. Previous studies investigating social influence on risky decision making have focused mainly on the description-based task where information sampling from experience does not play any important role in decision making. In contrast, our focus here is on the experience-based (i.e., learning based) risky decision making where information sampling processes are responsible for the proximate causes of risk-aversion, whose mechanisms can be independent from the utility function-based risk sensitivity measured in the description-based task. This distinction is important, because the very nature of information sampling in the experienced-based task plays the core role in both the hot stove effect and the collective rescue effect. To make this point clear, we have added sentences in the Introduction as follows:

–(Lines 72 – 82): How, if at all, can group-living animals improve collective decision accuracy while suppressing the potentially deleterious constraint of decision-making biases through trial-and-error learning? One of the strong candidates of explaining this gap is the fact that studies in human social learning in risky decision making have focused only on either the description based gambles (Chung et al., 2015; Bault et al., 2011; Suzuki et al., 2016; Shupp and Williams, 2008) or extreme conformity where individual choices are regulated fully by others’ behaviour (Denrell and Le Mens, 2007, 2016), but not on experienced-based situations where both individual and social learning affect behavioural outcomes, a form of decision making widespread in group-living animals and humans (Hertwig and Erev, 2009; Camazine et al., 2001; Toyokawa et al., 2019).

One potentially interesting design would be to test people in a situation where only the minority of the group members choose the optimal option (riskier option). If participants' choices become riskier even in this condition, we can conclude that they were not just copying the majority, but were maximising their reward by observing others' decisions and outcomes.

The situation described by the reviewer here is exactly what happened in our results. Risk-aversion was mitigated not because the majority chose the risky option, nor were individuals simply attracted towards the majority. Rather, participants’ choices became risker even though the majority chose the safer alternative at the outset. The mechanism behind such an ostensibly ‘minority effect’ is explained in the main text as follows, and we now highlight more clearly what this means:

– (Line 516 – 526): Despite conformity, the probability of choosing the suboptimal option can decrease from what is expected by individual learning alone. Indeed, an inherent individual preference for the safe alternative, expressed by the softmax function $e^{\beta Q_{s}}/(e^{\beta Q_{s}}+e^{\beta Q_{r}})$, is always mitigated by the conformist influence $N_{s}^{\theta }/(N_{s}^{\theta }+N_{r}^{\theta })$ as long as the former is larger than the latter. In other words, risk-aversion was mitigated not because the majority chose the risky option, nor were individuals simply attracted towards the majority. Rather, participants’ choices became risker even though the majority chose the safer alternative at the outset. Intuitively, under social influences (either because of informational or normative motivations), individuals become more explorative, likely to continue sampling the risky option even after he/she becomes disappointed by poor rewards.

In this study the authors investigated the effect of social influence on individuals' risk aversion in a 2-armed bandit task. Using a reinforcement learning model and a dynamic model they showed that social influence can in fact diminish risk aversion. The authors then conducted a series of online experiments and observed the same effect in their experimental data as well. The research question is timely, and the modelling has been done carefully. However, I have some comments and concerns about the interpretations of their findings.

– My first question is do the participants copy others because the other risky option sounds better in terms of reward or because they copy others just because being in alignment with others is rewarding? This brings us to the distinction between informational and normative influence. For example, a recent study showed that copying others is not necessarily motivated by maximising accuracy (Mahmoodi et al. 2018, see also Cialdinin and Goldstein 2004). In their experimental data, the authors found that participants do not copy others (choosing the risky options) as much as they should do. Does it suggest that their conformity toward others cannot be fully explained by informational motives (where the aim of conformity is to maximise payoff/accuracy). I suggest that the authors discuss each of these possibilities and then explain to which of these two types of influence their findings belong to.

We agree that two different motivations for conformity might have played a role in our experimental setup, although we did not explicitly distinguish these factors in our theoretical development. We have discussed further on this point and edited some texts in the Discussion as we have shown in our response to the reviewer 1 above.

– An earlier study showed that people's decisions become riskier when they make decisions with others (Bault et al. PNAS 2011). Could this explain the findings that are presented in this paper? Can the models distinguish between these two types of change in behaviour? I strongly suggest the authors to discuss the Bault et al. paper and discuss how their findings deviate from this study.

We have added texts explaining the relationship between our study and other studies using the description-based gambling tasks such as Bault et al. (2011), as we have shown in our response to the reviewer 2 (see above). In general, Bault et al. (2011) focuses on the description-based task where individuals can access to the profile of gambles, whereas our focus is on the experience-based decision making where information sampling through choices is crucial. The fact that previous human collective risky decision-making studies have been dominated mostly by the description-based gambling seems to account for the ostensible gap between maladaptive collective illusion reported in human conformity studies and collective intelligence documented in animal conformity studies. Since information sampling through experience is the crucial factor in our results, the rescue effect would never emerge if we used the description-based tasks.

Another key difference between our model and Bault et al. (2011) was whether others’ payoff information was available or not. Bault et al. focused on the situation where participants could see others’ payoffs, hence assuming the richer social information transmission than what assumed by our frequency-based social learning model. The implications of this difference were discussed in detail as follows:

– (Lines 600 – 609): Information about others’ payoffs might also be available in addition to inadvertent social frequency cues in some social contexts (Bault et al., 2011), especially with the aid of online communication tools or benevolent pedagogical acts from others. Although communicative acts may transfer information about behavioural alternatives that one has never tried before and may inform about forgone payoffs from other alternatives, which could mitigate the hot stove effect (Denrell, 2007; Yechiam and Busemeyer, 2006), it may further amplify the suboptimal decision bias if information senders, despite their cooperative motivation, selectively filter out some pieces of information they think are redundant (Moussaïd et al., 2015).

– In one section the authors show that reducing heterogeneity in groups undermines group performance. It brought my attention to a study (Lorenz et al. PNAS 2011) which suggested that social influence can undermine wisdom of crowds through reducing heterogeneity of opinions. It seems that the authors are presenting the same phenomenon as that suggested by Lorenz and colleagues. I suggest that the authors cite that study and discuss how their results is related to that study and whether their findings broaden our understanding of the effect of heterogeneity on collective performance.

Thank you for asking us to clarify the relation to this important study. We now explain explicitly why the collective rescue effect we find cannot be explained by the monotonic relationship between diversity and the wisdom of crowds (as it occurred in Lorenz et al., 2011). The followings are the discussion that we added:

– (Lines 505 – 513): Neither the averaging process of diverse individual inputs nor the speeding up of learning could account for the rescue effect. The individual diversity in the learning rate (α) was beneficial for the group performance, whereas that in the social learning weight (σ) undermines the average decision performance, which could not be explained simply by a monotonic relationship between diversity and wisdom of crowds (Lorenz et al., 2011). Self-organisation through collective behavioural dynamics emerging from the experience-based decision making must be responsible for the seemingly counter-intuitive phenomenon of collective rescue.

– Nothing can be found about the model in the main text. Similarly, some of the terms are not even defined before they are used in the main text. For example, the term "asocial learning" is only defined in the Figure 2 caption. I suggest that the authors briefly explain the model and the key terms in the main text before presenting the result. I also strongly suggest that the authors mention in the main text that the detail of the model is presented in the methods.

We now include ‘the Agent-Based Model’ section right after the Introduction (line 94 – 196), explaining the details of both task setups and the reinforcement learning models. We introduce the term ‘asocial learning’ in this new section as follows:

– (Lines 188 – 196): Note that, when σ = 0, there is no social influence, and the decision maker is considered as an asocial learner. It is also worth noting that, when σ = 1 with θ > 0, individual choices are assumed to be contingent fully upon majority's behaviour, which was assumed in some previous models of strong conformist social influences in sampling behaviour (Denrell and LeMens, 2016). Our model is a natural extension of both the asocial reinforcement learning and the model of ‘extreme conformity’, as these conditions can be expressed as a special case of parameter combinations. We will discuss the implications of this extension in the Discussion. The descriptions of the parameters are shown in Table 1.

We mention that the details of the dynamics model and that of the online experiments are presented in the Method as follows:

– (Line 329): The full details of this dynamics model are shown in the Method and Table 3.

– (Lines 436 – 439): The experimental task was basically a replication of the agent-based model described above, although the parameters of the bandit tasks were different (see the Method for the details of the experimental procedures; Supplementary Figure 11).

In the introduction reads: social influence does not mindlessly increase risk seeking; instead, it may work only when to do so is adaptive. I believe this sentence is vague in its current form. I suggest that the authors elaborate on it, especially on the last part (i.e. to do so is adaptive).

To clarify this point, we have changed the sentence as follows:

– (Lines 208 – 211): Interestingly, such a switch to risk seeking did not emerge when risk aversion was actually optimal (Supplementary Figure 9), suggesting that social influence does not always increase risk seeking; instead, the effect seems to be more prominent especially when risk seeking is beneficial in the long run.

Reviewer #2:

The paper considers an interesting puzzle. While most psychological studies of conformity tend to focus on negative effects, animal research highlights positive effects of conformity. The current analysis tries to clarify this apparent puzzle by clarifying one positive effect of conformity: Reduction of the hot stove effect that impairs maximization when taking risk is optimal.

The paper can be improved by building on previous efforts (e.g., Denrell and Le Mens, 2007) to clarify the impact of social influence on the hot stove effect. It would also be good to try to simplify the model, and use the same tasks in the theoretical analysis and the experimental study.

We agree to the reviewer’s point that our theory could become more impactful by relating to previous models such as Denrell & Le Mens (2007; 2016). We have explained what the critical difference between our model and the previous model is, highlighting how our model can be considered as a natural extension of previous conformity models. Notably, our model includes the cases explored by Denrell & Le Mens (2016) as an extreme setting of the social learning parameters where individual decision making is regulated fully by conformist social influence (that is,σ=1 andθ>1 for all individuals). Although the minor technical details between ours and their model are not identical, we were indeed able to replicate the pattern they found (i.e., the collective illusion by copying the majority’s behaviour) especially when social learning parameters (i.e., σandθ) were very high. The texts we added are as follows:

–(Lines 188 – 196): “Note that, when σ = 0, there is no social influence, and the decision maker is considered as an asocial learner. It is also worth noting that, when σ = 1 with θ > 0, individual choices are assumed to be contingent fully upon majority's behaviour, which was assumed in some previous models of strong conformist social influences in sampling behaviour (Denrell & LeMens, 2016). Our model is a natural extension of both the asocial reinforcement learning and the model of ‘extreme conformity’, as these conditions can be expressed as a special case of parameter combinations. We will discuss the implications of this extension in the Discussion. The descriptions of the parameters are shown in Table 1.”

To keep the model as simple as possible, we have added only two parameters for the frequency-based social learning processes, namely, σ(copying weight) and θ(conformity exponent). Previous studies have established that these two processes (i.e., the rate of social learning and the strength of conformity) affect collective dynamics differently (e.g., Kandler & Laland, 2013; Toyokawa et al., 2019). Therefore, we must consider these two parameters explicitly. We could have made our model more complex and more realistic by, for instance, considering temporally changing social influences (Toyokawa et al., 2019; Deffner et al., 2020), which we believe is worth exploring in the future studies. However, we aimed to limit our analysis to the simplest case so as to connect the literature on reinforcement learning and the hot stove effect (Denrell, 2007).

The discrepancy between our theoretical model and the online experimental tasks has by now been resolved by additional simulations shown in Supplementary Fig. 11 (Figure 1 – figure supplement 5). Here, we have shown that the rescue effect emerges robustly across different settings of the bandit tasks used in the online experiment. The reason why we focused on the Gaussian distribution task in the main result of the Agent-Based Model section was for the sake of mathematical tractability. The Gaussian task was theoretically well established, and its analytical solution was available (Denrell, 2007), which made our findings much clearer because we can assure that performance of social learners deviates truly from the analytical solution of asocial reinforcement learners’ performance (Fig 1).

One interesting open question involves the impact of the increase in the information, available today (in social networks like Facebook) concerning the behavior of other individuals. I think that the current analysis predicts an increase in risk taking.

We have added some discussion on this issue in the discussion section as follows:

– (Lines 600 – 609): “Information about others’ payoffs might also be available in addition to inadvertent social frequency cues in some social contexts (Bault et al., 2011), especially with the aid of online communication tools or benevolent pedagogical acts from others. Although communicative acts may transfer information about behavioural alternatives that one has never tried before and may inform about forgone payoffs from other alternatives, which could mitigate the hot stove effect (Denrell, 2007; Yechiam and Busemeyer, 2006), it may further amplify the suboptimal decision bias if information senders, despite their cooperative motivation, selectively filter out some pieces of information they think are redundant (Moussaïd et al., 2015).”

The paper considers an interesting puzzle. While most psychological studies of conformity tend to focus on negative effects, animal research highlights positive effects of conformity. The current analysis tries to clarify this apparent puzzle by clarifying one positive effect of conformity: Reduction of the hot stove effect that impairs maximization when taking risk is optimal.

The paper's main shortcoming is the fact that it is difficult to understand how it adds to the observations presented in Denrell and Le Mens (2007), and more recent research by Le Mens. It is possible that the authors can address this shortcoming by clarifying the difference between pure conformity (or imitation) and the impact of social influence examined by Le Mens and his co-authors.

Denrell and Le Mens (2007 and 2016) are indeed very relevant to our topic. We cite these two papers in our revised manuscript. Their 2007 paper considered opinion dynamics of a pair of individuals, while their 2016 paper extended it to multiple players (n≧2) that is more relevant to our model. The most crucial difference between their 2016 model and ours is that whilst they only considered a very strong conformity bias whereby individual choices were determined fully by other people’s opinion state, we have considered a wider range of conformist social influences from extremely weak (σ = 0; asocial reinforcement learning) to extremely strong (σ = 1; akin to the strong conformity assumed in Denrell and Le Mens (2016)). As we showed in our results, this relaxation of allowing the intermediate-level of conformist social influence in decision making is the necessary condition to generate the collective rescue effect. To clarify this point, we have modified several texts as follows:

– (Lines 72 – 82): “How, if at all, can group-living animals improve collective decision accuracy while suppressing the potentially deleterious constraint of decision-making biases through trial-and-error learning? One of the strong candidates of explaining this gap is the fact that studies in human social learning in risky decision making have focused only on either the description-based gambles (Chung et al., 2015; Bault et al., 2011; Suzuki et al., 2016; Shupp and Williams, 2008) or extreme conformity where individual choices are regulated fully by others’ behaviour (Denrell and Le Mens, 2007, 2016), but not on experienced-based situations where both individual and social learning affect behavioural outcomes, a form of decision making widespread in group-living animals and humans (Hertwig and Erev, 2009; Camazine et al., 2001; Toyokawa et al., 2019).”

– (Lines 188 – 196): “Note that, when σ = 0, there is no social influence, and the decision maker is considered as an asocial learner. It is also worth noting that, when σ = 1 with θ > 0, individual choices are assumed to be contingent fully upon majority's behaviour, which was assumed in some previous models of strong conformist social influences in sampling behaviour (Denrell and LeMens, 2016). Our model is a natural extension of both the asocial reinforcement learning and the model of ‘extreme conformity’, as these conditions can be expressed as a special case of parameter combinations. We will discuss the implications of this extension in the Discussion. The descriptions of the parameters are shown in Table 1.”

– (Lines 288 – 292): “This was because individuals with lower σ could benefit less from social information, while those with higher relied so heavily on social frequency information that behaviour was barely informed by individual learning, resulting in maladaptive herding or collective illusion (Denrell and Le Mens, 2016; Toyokawa et al., 2019).”

– (Lines 495 – 504): “We have demonstrated that frequency-based copying, one of the most common forms of social learning strategy, can rescue decision makers from committing to adverse risk aversion in a risky trial-and-error learning task, even though a majority of individuals are potentially biased towards suboptimal risk aversion. Although an extremely strong reliance on conformist influence can raise the possibility of getting stuck on a suboptimal option, consistent with the previous view of herding by conformity (Raafat et al., 2009; Denrell and Le Mens, 2016), the mitigation of risk aversion and the concomitant collective behavioural rescue could emerge in a wide range of situations under modest use of conformist social learning.”

– (Lines 557 – 560): “Such the synergistic interaction between positive and negative feedback could not be predicted by the collective illusion models where individual decision making is determined fully by the majority influence because no negative feedback would be able to operate.”

Another shortcoming involves the difference between the choice task analyzed in the theoretical analysis, and the task examined in the experiment. The theoretical analysis focuses on normal distributions, and the experiment focuses on asymmetric bimodal distributions. The authors suggest that they chose to switch to asymmetric bimodal distributions as the hot stove effect, exhibited by human subjects, in the case of normal distributions is not strong. If this is the case, it would be good to adjust the theoretical model and use a model that better capture human behavior.

We conducted additional simulations using the same bandit task setups as we used in the online experiment, confirming that the results do not change across conditions. Please find the details of this point in our reply to the review from reviewer #2 above.

A third shortcoming involves the complexity to the theoretical model. Since this model is only used to demonstrate that conformity can reduce the hot stove effect, and is not supposed to capture the exact magnitude of the two effects, I could not understand why it includes so many parameters. For example, it would be nice to add only one parameter to the basic reinforcement learning model. If more parameters are needed it would be good to show why they are needed.

As we discussed in the reply to the above, we agree that we should restrict the model to be as simple as possible. We believe that the current modelling is one of the simplest forms that can capture both the reliance on social influence (captured by *σ*) and the strength of conformity (captured by *θ*) separately.

Reviewer #3:

The authors use reinforcement learning and dynamic modeling to formalize the favorable effects of conformity on risk taking, demonstrating that social influence can produce an adaptive risk-seeking equilibrium at the population level. The work provides a rigorous analysis of a paradoxical interplay between social and economic choice.

Conformity is commonly attributed to either an intrinsic reward of group membership, or to inferences about the optimality of others' behavior (i.e., normative vs. informational). Neither of these aspects of conformity are addressed here, which limits the interpretability of the results. For example, if there is an intrinsic reward associated with majority alignment, that should contribute to the reinforcement of such decisions; moreover, inferences about the optimality of observed behavior likely change from early trials, in which others can be assumed to simply explore, to later trials, in which the decisions of others may be indicative of their success. The work would be more impactful if it considered how these factors might affect the potential for collective rescue.

An interesting question is whether a substantial payoff contingent on choosing a risky option may server to reinforce the act of risk taking itself, and how such processes might propagate social influence across environments.

I suspect that the paper was initially written with the Methods following the Introduction, and with the Methods being subsequently moved to the end without much additional editing. As a result, very few of the variables and concepts (e.g., conformist influence, copying weight, positive/negative feedback) are defined in the main text upon first mention, which makes for extremely onerous reading.

We have substantially revised the structure of the manuscript, now placing the ‘a Agent-Based Model’ section between the Introduction and the Results. In the current form, all key parameters (namely, conformist influence and copying weight) as well as other important concepts (e.g., positive feedback) are defined when it first appears:

– (Line 61 – 64): “Given that behavioural biases are ubiquitous and learning animals rarely escape from them, it may seem that conformist social influences may often lead to suboptimal herding or collective illusion through recursive amplification of the majority influence (i.e., positive feedback)”

Also, we deleted the term ‘negative feedback’ from the Introduction so that the term first appears at line 412 so that the meaning becomes clearer:

– (Lines 410 – 413): “Crucially, the reduction of $Ps^{–}$^−^ leads to further reduction of $Ps^{–}$^−^ itself through decreasing $Ns^{–}$, thereby further decreasing the social influence supporting the safe option $N_{s}^{\theta }/(N_{r}^{\theta }+N_{s}^{\theta })$. Such a negative feedback process weakens the concomitant risk aversion.”

Cases where the risky option yields a lesser mean payoff, producing a potentially detrimental social influence, should be given full weight in the main text, and should have been included in the behavioral study. Generally, the discrepancy between modeling and behavioral results is a bit disappointing. It is unclear why the behavioral experiment was not designed so as create the most relevant conditions.

To address this important point, we have conducted an additional series of experiments in which the risky option yields a smaller mean payoff than the safe alternative (namely, the negative risk premium [NRP] task), and report on it both in the theoretical part (see Supplementary Figure 18 [Figure 6 —figure supplement 2]) as well as in the experimental part (Figure 6 and Table 2; see also Supplementary Figure 19). In general, the model prediction was supported by the data from the NRP condition, suggesting that social influences could slightly be detrimental in such a condition because promotion of exploration increased the suboptimal risk taking. Nevertheless, the extent to which risk taking was increased by social influence in the NRP task was smaller than the extent to which optimal risk taking was increased in the positive risk premium tasks. Also, a previous study found that risk and reward are often correlated positively in many real-life circumstances (Pleskac and Hertwig, 2014), suggesting that situations where social influence is detrimental might be less common than situations where social influence is beneficial. Therefore, our conclusion that conformist social learning is more likely to promote adaptive risk taking should widely hold.

To highlight the results from these additional analyses and experiments, we have modified several parts of our texts as follows:

– (Lines 434 – 445): “To investigate whether the collective rescue effect can operate in reality, we conducted a series of online behavioural experiments using human participants. The experimental task was basically a replication of the agent-based model described above, although the parameters of the bandit tasks were different (see the Method for the details of the experimental procedures; Supplementary Figure 11). One hundred eighty-five adult human subjects performed the individual task without social interactions, while 400 subjects performed the task collectively with group sizes ranging from 2 to 8 (Supplementary Figure 17 and 19). We used four different settings for the multiarmed bandit tasks. Three of them were positive risk premium (PRP) tasks that had an optimal risky alternative, while the other was a negative risk premium (NRP) task that had a suboptimal risky alternative (see Methods). l (Lines 446 – 455): The behavioural results with statistical model fitting confirmed the predictions of the theoretical model. In the PRP task subjects who had a larger estimated value of the susceptibility to the hot stove effect (*α_i_*(*β_i_* + 1)) were less likely to choose the risky alternative, whereas those who had a smaller value of *α_i_*(*β_i_* + 1) had a higher chance of choosing the safe alternative (Figure 6a–c), consistent with the theory of the hot stove effect (Figure 2, Supplementary Figure 11). In the NRP task, individuals tended to choose the favourable safe option more often than they chose the risky option in a range of the susceptibility value *α_i_*(*β_i_* + 1) (Figure 6d), which was also consistent with the model prediction (Supplementary Figure 18).”

– (Lines 480 – 493): “In the NRP task, conformist social influence undermined the proportion of choosing the optimal safe option and increased adverse risk seeking, although a complete switch of the majority's behaviour to the suboptimal risky option did not happen (Figure 6d; Suppelementary Figure 19). Such promotion of suboptimal risk taking was particularly prominent when the susceptibility value *α_i_*(*β_i_* + 1) was large. Nonetheless, the extent to which risk taking was increased in the NRP condition was smaller than that in the PRP tasks, consistent with our model prediction that conformist social learning is more likely to promote favourable risk taking (Supplementary Figure 18). It is worth noting that the estimated learning rates (i.e., mean *α_i_* = 0.48) in the NRP task were larger than that in other PRP tasks (mean *α_i_* < 0.21; Table 2), making social learning particularly deleterious when risk taking is suboptimal (Supplementary Figure 18). In the discussion, we will discuss about the effect of experimental setting on the human learning strategies, which can be explored in the future studies.”

– (Lines 561 – 571): “Through online behavioural experiments using a risky multi-armed bandit task, we have confirmed our theoretical prediction that simple frequency-based copying could mitigate risk aversion that many individual learners, especially those who had higher learning rates and/or lower exploration rates, would have exhibited as a result of the hot stove effect. The mitigation of risk aversion was also observed in the NRP task, in which social learning slightly undermined the decision performance. However, because riskiness and expected reward are often positively correlated in a wide range of decision-making environments in the real world (Pleskac and Hertwig, 2014), the detrimental effect of reducing optimal risk aversion when risk premium is negative could be negligible in many ecological circumstances, making the conformist social learning beneficial in most cases.”

My greatest concern is that the work does not integrate properly with its theoretical and empirical context. Additional analyses assessing the relative contributions of normative and informational conformity to socially induced risk-seeking would be helpful.

We have conducted additional simulations with the bandit task setting identical to the experimental tasks (see Supplementary Figure 11 (Figure 1 —figure supplement 5) for the PRP tasks; and Figure 18 (Figure 6 —figure supplement 2) for the NRP task). We have confirmed that our theoretical results can hold robustly across the range of different task settings, strengthening the implication of the finding.

We are fully aware of the important distinctions between normative and informational motivations underlying the use of social information. We have explicitly mentioned our stance at lines 159 – 167 that we limited our analysis to the informational context and suggested some future directions in the discussion at lines 572 – 586, as follows:

– (Line 159 – 167): “A payoff realised was independent of others’ decisions and it was drawn solely from the payoff probability distribution specific to each alternative, thereby we assume neither direct social competition over the monetary reward (Giraldeau and Caraco, 2000) nor normative pressures towards majority alignment (Cialdini and Goldstein, 2004; Mahmoodi et al., 2018). The value of social information was assumed to be only informational (Nakahashi, 2012). Nevertheless, our model could apply to the context of normative social influences, because what we assumed here was modifications in individual choice probabilities due to social influences, irrespective of underlying motivations of conformity.”

– (Lines 572 – 586): “The weak reliance on social learning, which affected only about 15% of decisions, was unable to facilitate strong positive feedback. The little use of social information might have been due to the lack of normative motivations for conformity and to the stationarity of the task. In a stable environment, learners could eventually gather enough information as trials proceeded, which might have made them less curious about information gathering including social learning (Rendell et al., 2010). In reality, people might use more sophisticated social learning strategies whereby they change the reliance on social information flexibly over trials (Deffner et al., 2020, Toyokawa et al., 2017, Toyokawa et al., 2019). Future research should consider more strategic use of social information, and will look at the conditions that elicit heavier reliance on the conformist social learning in humans, such as normative pressures for aligning with majority, volatility in the environment, time pressure, or an increasing number of behavioural options (Muthukrishna et al., 2015), coupled with larger group sizes (Toyokawa et al., 2019).”

[Editors’ note: what follows is the authors’ response to the second round of review.]

Essential revisions:

The manuscript has been improved, and Reviewer 2 recommends acceptance at this point, but Reviewer 3 has some remaining concerns, summarized below. We invite you to address all remaining concerns in a second round of revisions. Make sure to include point-by-point replies to each of Reviewer 3's recommendations.

1) Although the organization and writing is improved, it has some ways to go before the manuscript is ready for publication. For example, the basic aims and methods should be stated in one or two sentences at the end of the first, or at most second, paragraph of the introduction, giving the reader a clear sense of where things are going. Moreover, the "Agent-based model" section should be shortened (to include only what is needed to conceptually understand the model, leaving details for a table and the methods) and better integrated with the introduction, rather than inserted as (what appears to be) a super-section.

We thank the editors and the reviewers for this valuable suggestion. We totally agree with the value of giving readers a clear sense of the article’s structure in the beginning of the introduction. To do this, among addressing the other points related to the Introduction (see below), we have revised the Introduction sentence-by-sentence, and have made a central question and background of this paper much clearer in the first two paragraphs. Particularly, the aim of this paper is summarised at the end of the second paragraph the Introduction:

– Lines 60 – 63: “A theory that incorporates dynamics of trial-and-error learning and the learnt risk aversion into social learning is needed to understand the conditions under which collective intelligence operates in risky decision making.”

Also, we have deleted the subsection “Agent-based model” and integrated its contents into the end of the Introduction and the beginning of the Result. In both places, we defined technical terms as soon as they first appeared, and verbally described the concept and assumptions of the computational model. Please see the subsections “The decision-making task”, “The baseline model”, and “The conformist social influence model” in the Result section.

2) Just as the intro should include a concise description of the model, it should highlight the online experiments, and how they relate to the modeling.

Highlighting the online experiment and articulating the relationship between the experiment and models in the Introduction is a wonderful suggestion. We have modified the Introduction to make the aim of the experiment clear. The modified text is as follows:

– Lines 122 – 130: “Finally, to investigate whether the assumptions and predictions of the model hold in reality, we conducted a series of online behavioural experiments with human participants. The experimental task was basically a replication of the task used in the agent-based model described above, although the parameters of the bandit tasks were modified to explore wider task spaces beyond the simplest two-armed task. Experimental results show that the human collective behavioural pattern was consistent with the theoretical prediction, and model selection and parameter estimation suggest that our model assumptions fit well with our experimental data.”

3) The result section should start with a paragraph outlining the various hypotheses and corresponding analyses (i.e., a "roadmap" for the section).

We thank the editors for this insightful suggestion. Sketching a roadmap before showing detailed results is a great idea. To guide readers smoothly from the Introduction to the Result, we have outlined an overview of our analysis at the end if the Introduction:

– Lines 109 – 132: “In the study reported here, we firstly examined whether a simple form of conformist social influence can improve collective decision performance in a simple multi-armed bandit task using an agent-based model simulation. We found that promotion of favourable risk taking can indeed emerge across different assumptions and parameter spaces, including individual heterogeneity within a group. This phenomenon occurs thanks, apparently, to the non-linear effect of social interactions, namely, *collective behavioural rescue*. To disentangle the core dynamics behind this ostensibly self-organised process, we then analysed a differential equation model representing approximate population dynamics. Combining these two theoretical approaches, we identified that it is a combination of positive and negative feedback loops that underlies collective behavioural rescue, and that the key mechanism is a promotion of information sampling by modest conformist social influence.

Finally, to investigate whether the assumptions and predictions of the model hold in reality, we conducted a series of online behavioural experiments with human participants. The experimental task was basically a replication of the task used in the agent-based model described above, although the parameters of the bandit tasks were modified to explore wider task spaces beyond the simplest two-armed task. Experimental results show that the human collective behavioural pattern was consistent with the theoretical prediction, and model selection and parameter estimation suggest that our model assumptions fit well with our experimental data. In sum, we provide a general account of the robustness of collective intelligence even under systematic risk aversion and highlight a previously overlooked benefit of conformist social influence.”

As we believe that repeating the general outline at the beginning of the result section would be redundant, we put short introductory sentences at the beginning of each subsection in the Result. Especially, we have made our experimental hypotheses clearly stated at the beginning of the “Experimental demonstration” subsection as follows:

– Lines 507 – 512: “On the basis of both the agent-based simulation (Figure 1 and Supplementary Figure 9) and the population dynamics (Figure 5 and Supplementary Figure 16), we hypothesised that conformist social influence promotes risk seeking to a lesser extent when the RP is negative than when it is positive. We also expected that whether the collective rescue effect emerges under positive RP settings depends on learning parameters such as $\alpha_{i}(\beta_{i}+1)$ (Supplementary Figure 11d–f).”

4) Please quantify the performance of your model relative to others with formal comparisons (e.g., Bayesian Model Selection).

We really appreciate this insightful suggestion. Thanks to the formal model comparison using the Bayesian model selection based on WAIC, our finding has been much strengthened. We have now included both the model recovery check and the model comparison result in the new Supplementary Figure 18 (Figure 6 —figure supplement 2) in page 54. The successful model recovery has ensured that the hierarchical Bayesian model fitting method could reliably differentiate between the candidate models, and we confirmed that the model comparison favoured the decision-biasing model that we used in our main analysis. We have added some texts to include this finding as follows:

– Lines 277 – 286: “Further, the conclusion still held for an alternative model in which social influences modified the belief-updating process (the value-shaping model; Najar et al., 2020) rather than directly influencing the choice probability (the decision-biasing model) as assumed in the main text thus far (see Supplementary Methods; Supplementary Figure 8). One could derive many other more complex social learning processes that may operate in reality; however, the comprehensive search of possible model space is beyond the current interest. Yet, decision biasing was found to fit better than value shaping with our behavioural experimental data (Supplementary Figure 18), leading us to focus our analysis on the decision-biasing model.”

– Lines 513 – 517: “The Bayesian model comparison (Stephan et al., 2009) revealed that participants in the group condition were more likely to employ decision-biasing social learning than either asocial reinforcement learning or the value-shaping process (Supplementary Figure 18). Therefore, in the following analysis we focus on results obtained from the decision-biasing model fit.”

– Line 950 – 956: “We compared the baseline reinforcement learning model, the decision biasing model, and the value-shaping model (see Supplementary Methods) using Bayesian model selection (Stephan et al., 2009). The model frequency and exceedance probability were calculated based on the Widely Applicable Information Criterion (WAIC) values for each subject (Watanabe and Opper, 2010). We confirmed accurate model recovery by simulations using our task setting (Supplementary Figure 18).”

5) Please quantify all claims of associations with effect sizes and clearly justify all parameter cut-offs/values.

6) Streamline figures and included predicted/observed result plots wherever possible.

We thank the editors and the reviewers for this great suggestion. Having conducted an additional data analysis using a standard GLMM with a hierarchical Bayesian estimation method, we have quantified all the empirical findings with their effect sizes accompanied by the Bayesian credible intervals. Please see the new Table 3 (page 28) for the estimated coefficients of the GLMM as well as a new Supplementary Figure 17 (Figure 6 —figure supplement 1; page 53) for the prediction from the fit GLMM. Also, we deleted the arbitrary parameter cut-offs, and showed the effect of the copying weight in a continuous manner in the Figure 6 (page 26).

To streamline the two different types of empirical analyses (that are the fit computational model and the raw data with fit GLMM), we have separated them into the computational model prediction (Figure 6) and the GLMM regression with the experimental data (Supplementary Figure 17). The matched pattern between them supports that the fit computational model was able to reproduce the actual participants’ behaviour. To highlight these points, we have added a new paragraph in the result section as follows:

– Lines 548 – 560: “To quantify the effect size of the relationship between the proportion of risk taking and each subject’s best fit learning parameters, we analysed a generalised linear mixed model (GLMM) fitted with the experimental data (see Methods; Table 3). Within the group condition, the GLMM analysis showed a positive effect of on risk taking for every task condition (Table 3), which supports the simulated pattern. Also consistent with the simulations, in the positive RP tasks, subjects exhibited risk aversion more strongly when they had a higher value of $\alpha_{i}(\beta_{i}+1)$ (Supplementary Figure 17a–c). There was no such clear trend in data from the negative RP task, although we cannot make a strong inference because of the large width of the Bayesian credible interval (Supplementary Figure 17d). In the negative RP task, subjects were biased more towards the (favourable) safe option than subjects in the positive RP tasks (i.e., the intercept of the GLMM was lower in the negative RP task than in the others).”

Reviewer #3:

The authors showed that when individuals learn about how they should decide in a situation where they can choose between a risky and a safe option, they might overcome maladaptive biases (e.g. exaggerated risk-aversion when risk-taking would be beneficial) by conforming with group behaviour. Strengths include rigorous and innovative computational modeling, a weakness might be that the set-up of the empirical study did not actually widely provoke behavioral phenomena at question, e.g. social learning (which is arguably at the core of the research question). Even though I am reviewing a revised manuscript I would hope the authors find a way to further improve clarity in the presentation of their research question and results.

My main concern was that even in the revised version I read, I found the paper not as accessible as I think it should be for a wide readership of a journal like eLife; and often times I found that things you could communicate in a straightforward way are put too complicated/expressed very verbosely. When reading the previous reviews after having read the revised paper, I also got the feeling that there were some misunderstandings. You clarified these specific points well in your responses, I think, but the lack in clarity might be even more drastic with an interdisciplinary readership this journal aims at as compared to the experts the journal has recruited now for this review? I will try to give some examples below.

We thank the reviewer very much for this valuable suggestion. We fully agreed that the previous manuscript was not very accessible to a wider audience we aim to reach. We have revised the manuscript substantially to improve its clarity, accessibility, and rigor. Especially, both the Introduction and introductory paragraphs of the Result were rewritten to let them clearly articulate our research question and aims. In the following, we will explain, point-by-point, how we have revised the manuscript in responding to each of the reviewer’s concerns.

The introduction should, imho provide a general intro to the question of the paper and how you've arrived to ask that question, avoiding too much technical jargon. After having read the paper, I realized that the research question is pretty straight-forward (and interesting) and derived from 1/2 previous observations, but this didn't become clear on the very first read.

Just as an example, some of the first sentences are…

"One rationale behind this optimistic view might come from the assumption that individuals tend to prefer a behavioural option that provides larger net benefits in utility over those providing lower outcomes. Therefore, even though uncertainty makes individual decision-making fallible, statistical filtering through informational pooling may be able to reduce uncertainty by cancelling out such noise."

This is only 1 example (it is something I noted throughout the paper… and also came up in the previous round of reviews) where I think these 2 sentences require some if not a little more background in decision-making (net benefits, utility, uncertainty, noise, stat filtering, informational pooling) to be understandable.

We thank the reviewer for this valuable feedback. The sentence referred here was in the first paragraph of the Introduction of the previous manuscript, which was indeed not very accessible for many interdisciplinary readers. Having restructured the Introduction, we believe that the aims and motivations behind this study became clearer and selfexplanatory. The first paragraph of the Introduction is now read as follows:

– Lines 29 – 46: “Collective intelligence, a self-organised improvement of decision making among socially interacting individuals, has been considered one of the key evolutionary advantages of group living (Camazine et al., 2001; Krause and Ruxton, 2002; Sumpter, 2006; Ward and Zahavi, 1973). Although what information each individual can access may be a subject of uncertainty, information transfer through the adaptive use of social cues filters such ‘noises’ out (Laland, 2004; Rendell et al., 2010), making individual behaviour on average more accurate (Hastie and Kameda, 2005; King and Cowlishaw, 2007; Simons, 2004). Evolutionary models (Boyd and Richerson, 1985; Kandler and Laland, 2013; Kendal et al., 2005) and empirical evidence (Toyokawa et al., 2014, 2019) have both shown that the benefit brought by the balanced use of both socially and individually acquired information is usually larger than the cost of possibly creating an alignment of suboptimal behaviour among individuals by herding (Bikhchandani et al., 1992; Giraldeau et al., 2002; Raafat et al., 2009). This prediction holds as long as individual trialand-error learning leads to higher accuracy than merely random decision making (Efferson et al., 2008). Copying a common behaviour exhibited by many others is adaptive if the output of these individuals is expected to be better than uninformed decisions.”

Other terminology like e.g. collective illusion, opportunity costs, description-based vs. experienced-based risk-taking paradigms, frequency-based influences, would be nice to be either defined in the text or replaced by a more accessible description in the introduction.

I know it is sometimes hard to mentalize which terminology others not working on the same things might struggle with, but given that this is an interdisciplinary journal, might it perhaps make sense to ask a researcher friend who is not exactly working on this topic to give it a read?

We thank the reviewer so much for specifying these reader-unfriendly technical jargons. We have defined both “collective illusion” and “frequency-based” when they first appear, with the other terms eliminated from the text. Please see the revised manuscript listed below. We have asked several colleagues from different fields to read the manuscript, which we believe has made the manuscript much more accessible. The modified sentences are as follows:

– Lines 94 – 95: “a mismatch between the true environmental state and what individuals believed (’collective illusion’; Denrell and Le Mens, 2016).”

– Lines 65 – 71: “it may seem that social learning, especially the ’copy-the-majority’ behaviour (aka, ’conformist social learning’ or ’positive frequency-based copying’; Laland, 2004), whereby the most common behaviour in a group is disproportionately more likely to be copied (Boyd and Richerson, 1985), may often lead to maladaptive herding, because recursive social interactions amplify the common bias (i.e., a positive feedback loop; Denrell and Le Mens, 2007, 2016; Dussutour et al., 2005; Raafat et al., 2009).”

"such a risk-taking bias constrained by the fundamental nature of learning may function independently from the adaptive risk perception (Frey et al., 2017), potentially preventing adaptive risk taking."

Unclear without knowing or looking up the Frey paper – shorten ("to be too risk-averse might be maladaptive in some contexts"?) or explain.

We totally agreed that the sentence was unclear. Indeed, merely mentioning that risk aversion may be adaptive in some context (Real and Caraco, 1986; McNamara and Houston, 1992; Yoshimura and Clark, 1991), and that risk aversion may arise from different mechanisms (Frey et al. 2017), was not directly related to the focus of this paper. What we would like to highlight in the second paragraph of the Introduction was the omnipresent possibility of risk aversion arising from reinforcement learning. In the revised version, therefore, we concentrated on this focal point and deleted those irrelevant topics.

– Lines 47 – 63: “However, both humans and non-human animals suffer not only from environmental noise but also commonly from systematic biases in their decision making (e.g., Harding et al., 2004; Hertwig and Erev, 2009; Real, 1981; Real et al., 1982). Under such circumstances, simply aggregating individual inputs does not guarantee collective intelligence because a majority of the group may be biased towards suboptimization. A prominent example of such a potentially suboptimal bias is risk aversion that emerges through trial-and-error learning with adaptive information-sampling behaviour (Denrell, 2007; March, 1996). Because it is a robust consequence of decision making based on learning (Hertwig and Erev, 2009; Yechiam et al., 2006; Weber, 2006; March, 1996), risk aversion can be a major constraint of animal behaviour, especially when taking a high-risk high-return behavioural option is favourable in the long run. Therefore, the ostensible prerequisite of collective intelligence, that is, that individuals should be unbiased and more accurate than mere chance, may not always hold. A theory that incorporates dynamics of trial-and-error learning and the learnt risk aversion into social learning is needed to understand the conditions under which collective intelligence operates in risky decision making.”

Line 74-82 extremely long sentence – I think can easily be simplified? – "previous studies have neglected contexts where individuals learn about the environment both by own and others' experiences"

We agreed that the sentence was too long. Because this relates to the central motivation behind our choice of model and question, we elaborated it in two paragraphs as follows:

– Lines 83 – 99: “In this paper, we propose a parsimonious computational mechanism that accounts for the emerging improvement of decision accuracy among suboptimally riskaversive individuals. In our agent-based model, we allow our hypothetical agents to compromise between individual trial-and-error learning and the frequency-based copying process, that is, a balanced reliance on social learning that has been repeatedly supported in previous empirical studies (e.g., Deffner et al., 2020; McElreath et al., 2005, 2008; Toyokawa et al., 2017, 2019). This is a natural extension of some previous models that assumed that individual decision making was regulated fully by others’ beliefs (Denrell and Le Mens, 2007, 2016). Under such extremely strong social influence, exaggeration of individual bias was always the case because information sampling was always directed towards the most popular alternative, often resulting in a mismatch between the true environmental state and what individuals believed (’collective illusion’; Denrell and Le Mens, 2016). By allowing a mixture of social and asocial learning processes within a single individual, the emergent collective behaviour is able to remain flexible (Aplin et al., 2017; Toyokawa et al., 2019), which may allow groups to escape from the suboptimal behavioural state.”

– Lines 100 – 108: “We focused on a repeated decision-making situation where individuals updated their beliefs about the value of behavioural alternatives through their own action–reward experiences (experience-based task). Experience-based decision making is widespread in animals that learn in a range of contexts (Hertwig and Erev, 2009). The time-depth interaction between belief updating and decision making may create a non-linear relationship between social learning and individual behavioural biases (Biro et al., 2016), which we hypothesised is key in improving decision accuracy in self-organised collective systems (Camazine et al., 2001; Sumpter, 2006).”

Line 84-89 is really long, too.

I would have been interested to learn more about the online experiments in the intro.

We thank the reviewer very much for pointing this out. We totally agreed. In the revised version, we gave a more accessible roadmap of the paper at the end of the Introduction, rather than putting such a long-sentence summary. In this roadmap section, we have described more about the experiment and highlight the relationship between the theoretical models and the experiment. The revised paragraph of the Introduction is as follows:

– Lines 109 – 132: “In the study reported here, we firstly examined whether a simple form of conformist social influence can improve collective decision performance in a simple multi-armed bandit task using an agent-based model simulation. We found that promotion of favourable risk taking can indeed emerge across different assumptions and parameter spaces, including individual heterogeneity within a group. This phenomenon occurs thanks, apparently, to the non-linear effect of social interactions, namely, *collective behavioural rescue*. To disentangle the core dynamics behind this ostensibly self-organised process, we then analysed a differential equation model representing approximate population dynamics. Combining these two theoretical approaches, we identified that it is a combination of positive and negative feedback loops that underlies collective behavioural rescue, and that the key mechanism is a promotion of information sampling by modest conformist social influence.

Finally, to investigate whether the assumptions and predictions of the model hold in reality, we conducted a series of online behavioural experiments with human participants. The experimental task was basically a replication of the task used in the agent-based model described above, although the parameters of the bandit tasks were modified to explore wider task spaces beyond the simplest two-armed task. Experimental results show that the human collective behavioural pattern was consistent with the theoretical prediction, and model selection and parameter estimation suggest that our model assumptions fit well with our experimental data. In sum, we provide a general account of the robustness of collective intelligence even under systematic risk aversion and highlight a previously overlooked benefit of conformist social influence.”

I do understand the reasoning of copy and pasting the agent Based Model description after having read the previous reviews, but it confused me when reading the article first; I think it needs to be integrated with Intro/Results section better (the first paragraph reads like intro/background still, but then it is about the authors' current study). I fear that readers don't know where they are in the paper at that point. (I much prefer journals with the Methods-Results order rather than the one eLife uses, as this would naturally circumvent this problem, but that's the challenge here, I reckon.)

We totally agreed that the conceptual description of the method should be integrated in the Introduction and the Result. In the revised manuscript, we have elaborated the concept of the model using as an accessible language as possible. Especially, the new subsections “The decision-making task” (page 5), “The baseline model” (page 7), “The conformist social influence model” (page 8), as well as “The simplified population dynamics model” (page 16) have been substantially revised to have conceptual verbal descriptions about the assumption and formulation before showing detailed results.

Results

Subheadings: Make clearer which is the section that describes simulations and which is the empirical section.

Thank you for this valuable suggestion. We have now subheadings fully separated between the theoretical part and the experimental results, so that the empirical result is shown only in the subsection “An experimental demonstration” (page 21).

Can you maybe start with reiterating in a structured way which parameters you set to which values and why in the simulation before describing the effects of it, how many trials you simulate (you start speaking of elongated time horizons but do not mention the original horizon length other than in the figure legend?) etc?

We have added parameters used in the simulations before describing the result. The revision we made are as follows:

– Lines 146 – 147: “Unless otherwise stated, the total number of decision-making trials (time horizon) was set to T = 150 in the main simulations described below.”

– Line 301 – 307: “Individual values of a focal behavioural parameter were varied across individuals in a group. Other non-focal parameters were identical across individuals within a group. The basic parameter values assigned to non-focal parameters were α = 0.5, β = 7, σ = 0.3, and θ = 2, which were chosen so that the homogeneous group could generate the collective rescue effect. The groups’ mean values of the various focal parameters were matched to these basic values.”

I know the Najar work and I think it is cool that you can generalize your results also to a value-based framework, but I do not think readers that do not know the Najar study will be able to follow this at it is described now, which makes it more confusing than interesting. So, either elaborate what this means (accessible to non-initiated readers) or ban to the Supplement (would be a shame).

Would the value-based model fit the empirical data better?

Thank you so much for this valuable comment. As we described in our response to the editor’s point (4), we have conducted the Bayesian model comparison and have established that the decision-biasing model was likely to fit better than both the value-shaping model and the baseline reinforcement learning model. We have verbally described the value shaping model in the following paragraph, while the full details are shown in the supplementary method.

– Lines 277 – 286: “Further, the conclusion still held for an alternative model in which social influences modified the belief-updating process (the value-shaping model; Najar et al., 2020) rather than directly influencing the choice probability (the decision-biasing model) as assumed in the main text thus far (see Supplementary Methods; Supplementary Figure 8). One could derive many other more complex social learning processes that may operate in reality; however, the comprehensive search of possible model space is beyond the current interest. Yet, decision biasing was found to fit better than value shaping with our behavioural experimental data (Supplementary Figure 18), leading us to focus our analysis on the decision-biasing model.”

When reading the first part of the Results section I constantly wondered: How did the group behave / How was the behaviour of the group determined in the simulation? Was variability considered? (this is something that's been manipulated in some empirical studies building on descriptive risk scenarios, e.g. Suzuki et al). It becomes clear when reading on/looking at Figure 3, but it is such a crucial point that it needs to be made clear from the beginning.

This is a great suggestion and we agreed with the importance to consider the variability of individuals. To make this point clear in the Introduction, we have included this in the “roadmap” paragraph as follows:

– Lines 111 – 113: “We found that promotion of favourable risk taking can indeed emerge across different assumptions and parameter spaces, including individual heterogeneity within a group.”

I think it is a major limitation that in the empirical study actual social learning was extremely limited, given that the paper claims to provide a formal account of the function of social learning in this situation?…. I would have thought that indeed trying to provoke more use of social influence by altering the experimental setup in a way the authors propose in their discussion would have been important, and given that this can be done online, also a feasible option.

We thank the reviewer for pointing out the limitation of the current study. We totally agree that increasing the copying weight by experimental manipulations will indeed be an important future direction. As we discussed in the main text (lines 649 – 676), one of such a promising manipulation will be to use a ‘restless’ bandit task that is theoretically expected to induce both a higher learning rate and higher copying weight. Nevertheless, we believe that a direct link between the simplest form of theory (that is, a static bandit task) and experimental findings was a necessary first step toward developing further theoretical hypotheses in more complex settings. Therefore, in the current purpose we put the possibility of the restless bandit as a future task.

Also, empirically, susceptibility to the hot stove effect, i.e., *α*_*i*_(*β*_*i*_ + 1), seems to be very low – zero for most of the participants in some scenarios according to Figure 6A,B,C? – isn't this concerning, given that this is at the core of what the authors want to explain?

We thank the reviewer for raising this point. This is a wonderful question. The average value of the susceptibility to the hot stove effect across the conditions was about 0.2 ~ 0.6, but not zero (which was not visually obvious due to the scale of the x-axis, but can be derived from the fit parameter values shown in Table 2). Such a low level of the susceptibility to the hot stove effect was problematic in the 1-risky-1-safe task because asocial individuals were not likely to suffer from the hot stove effect (see Supplementary Figure 11a). Therefore, we conducted the other two positive RP 4-armed tasks where risk aversion was expected to emerge even if α (β +1) was as small as such values (Supplementary Figure 11b, c). However, as we have discussed in the main text, it is indeed an interesting future direction to manipulate both task and environment to elicit higher learning rate as well as heavier reliance on social learning.

"those with a higher value of the susceptibility to the hot stove effect (*α*_*i*_(*β*_*i*_ + 1)) were less likely to choose the risky alternative, whereas those who had a smaller value of *α*_*i*_(*β*_*i*_ + 1) had a higher chance of choosing the safe alternative (Figure 6a-c), " -- I'm confused- should it read "those who had a smaller value of *α*_*i*_(*β*_*i*_ + 1) had a higher chance of choosing the ‘risky’ alternative"?

Could you please quantify this correlation in terms of an effect size? The association is not linear, from the Figure? Please specify.

Is the association driven by α or by β or really by the product?

The reviewer is correct, the original sentence suggested the effect in the wrong, opposite direction. To quantify the effect size, we have conducted an additional GLMM, and the results are now read:

– Lines 548 – 560: “To quantify the effect size of the relationship between the proportion of risk taking and each subject’s best fit learning parameters, we analysed a generalised linear mixed model (GLMM) fitted with the experimental data (see Methods; Table 3). Within the group condition, the GLMM analysis showed a positive effect of on risk taking for every task condition (Table 3), which supports the simulated pattern. Also consistent with the simulations, in the positive RP tasks, subjects exhibited risk aversion more strongly when they had a higher value of $\alpha_{i}(\beta_{i}+1)$ (Supplementary Figure 17a–c). There was no such clear trend in data from the negative RP task, although we cannot make a strong inference because of the large width of the Bayesian credible interval (Supplementary Figure 17d). In the negative RP task, subjects were biased more towards the (favourable) safe option than subjects in the positive RP tasks (i.e., the intercept of the GLMM was lower in the negative RP task than in the others).”

The product, *α*_*i*_(*β*_*i*_ + 1), has been derived from the theoretical development of Denrell (2007) and has been used in our theoretical analysis too (Figure 2). Of course, the learning rate (α) and the inverse temperature (β) are a different free parameter that plays a different functional role in the learning algorithm. However, in the context of the hot stove effect, they play a correlated role, which allows us to compress a dimension in the analysis. Thanks to this, we can understand both theory (Figure 2) and empirical results (Figure 6 and Supplementary Figure 17) in a simple way. Therefore, for the sake of brevity, we believe that treating them as a product form *α*_*i*_(*β*_*i*_ + 1) is more straightforward for the current purpose than separating them apart.

"The behaviour in the group condition supports our theoretical predictions. In the PRP tasks, the proportion of choosing the favourable risky option increased with social influence (i) particularly for individuals who had a high susceptibility to the hot stove effect. On the other hand, social influence had little benefit for those who had a low susceptibility to the hot stove effect (e.g., *α*_*i*_(*β*_*i*_ + 1) {less than or equal to} 0.5)." Can you quantify this with statistically (effect sizes etc)?

We thank the reviewer very much for suggesting us to formally quantify the effect sizes. As we described above, we have conducted a GLMM analysis, and confirmed that the pattern emerged in the experiment matched well with the prediction of the calibrated computational model. We believe that our findings have been made more convincing by this additional analysis.

Did you do any form of model selection on the empirical data (with different set ups of your models, the reduced models (e.g. without σ), or the Najar type of model) to demonstrate that it is really your theoretically proposed model that fits the data best (e.g. Bayesian Model Selection)? Please include in the main manuscript. See Palminteri, TiCS on why this might be important.

I think Figure 6 is really overloaded. The legend somehow looks as it would belong only to panel c? The coloured plots are individual data points as a function of group size (which is what?) or copying weight? For me, in the current formatting, dots were too small to detect a continuous colour coding scheme (only yellow vs purple). The solid lines are simulated data? Can you show a regression line for the empirical data to allow for comparisons? Does it not differ substantially from the model predictions? I suggest making different plots for different purposes (compare predicted behaviour to empirical behaviour, show effect of copying weight, show effect of group size etc, show simulation were you plug in σ>0.4)

Thank you so much also for giving us this terrific suggestion. We have included both the model recovery test and model comparison based on Bayesian model selection in the main test (Supplementary Figure 18 [Figure 6 —figure supplement 2]) in page 54. Please see our response to the editor’s comment (4) for more details.

"In keeping with this, if we extrapolated the larger value of the copying of weight (i.e., $\bar{\sigma_{i}}$>0.4) into the best fitting social learning model with the other parameters calibrated, a strong collective rescue became prominent " – sorry, where does the value $\bar{\sigma }$>0.4 exactly come from for this analysis? Please give more detail/contextualise better.

This was indeed helpful feedback. As we described in our response to the editor’s comment (5) and (6), we have separated the computational model prediction and the data with a regression line into two figures. We have also included the varying σ in a gradual manner, varying across the range of individual fit σ for each task, rather than just showing an arbitrary high value (that was set to σ>0.4 in the previous manuscript). We believe that the current presentation of the empirical result allows readers to easily differentiate what was the data themselves and what was the model prediction.

Please try to be consistent with terminology *α*_*i*_(*β*_*i*_+ 1) is sometimes called 'susceptibility ' or 'susceptibility value', which might be confusing, given that in some published articles susceptibility refers to susceptibility to social influence which would be another parameter…. I suggest to go through the manuscript once more and strictly only use one term for each parameter (the one you introduce in the table).

Thank you so much again for your thorough review and insightful comments. We have gone through the text again and made all the terms consistent.
